# Adaptive Super-Twisting Tracking for Uncertain Robot Manipulators Based on the Event-Triggered Algorithm

**DOI:** 10.3390/s25051616

**Published:** 2025-03-06

**Authors:** Yajun Ma, Hui Zhao, Tao Li

**Affiliations:** 1 School of Electrical and Information Engineering, Tianjin University, Tianjin 300072, China; mayajun3690043@163.com; 2 Beijing Institute of Precision Mechatronics and Controls, Beijing 100076, China; 3 School of Electrical Engineering and Automation, Tianjin University of Technology, Tianjin 300384, China; 4 Beijing Research Center of Intelligent Equipment for Agriculture, Beijing Academy of Agriculture and Forestry Sciences, Beijing, 100097, China; mayj51062963@proton.me

**Keywords:** event-triggered control, adaptive super-twisting, robot manipulator, Zeno free execution

## Abstract

In this study, the authors present an event-triggered control scheme for uncertain robot manipulators combined with an adaptive super-twisting algorithm to handle uncertain robot manipulator systems with unknown external uncertainties and disturbances. The proposed controller can ensure the system-tracking performance while also guaranteeing the robust stability of the system. First, an event-triggered adaptive super-twisting control (ETASTC) method for multivariable second-order nonlinear systems is proposed. In addition, unlike the implementation of periodic control, in the event-triggered method, the control signal is updated by the requirement of system stability, thus avoiding the frequent periodic execution of control tasks. Furthermore, through rigorous proof, the Zeno free execution of the triggering sequence is also ensured. Lastly, the proposed method is illustrated through numerical simulation and experimental study, and the results show that the computational cost is saved while also ensuring the desired performance of the robot system.

## 1. Introduction

With the development of industrial technology, robot manipulators are widely used in various complex tasks, such as manufacturing, assembly, and aerospace exploration [1], which have successfully attracted the interest of scientists. At the same time, there is an increasing demand for the high-precision control of robot manipulators, mainly focusing on fast trajectory tracking accuracy, adaptive control, and stable convergence. A challenging topic regarding robot manipulators is how to deal with the robot dynamics in complex environments and in the presence of various interferences [2].

Therefore, achieving a better robot manipulator tracking performance in the presence of uncertain system parameters requires further research and improvement. Many advanced nonlinear control methods have been presented and are widely used in trajectory tracking control. For an uncalibrated camera–robot system with uncertainties, a novel global finite-time stability (FTS) [1] controller has been proposed, which can cope with rapid convergence problems. However, it is very difficult to design the control law accurately and select the appropriate control gain parameters. To overcome the problem of the high-accuracy tracking trajectory of a robot manipulator in the presence of uncertainties and external disturbances, a robust adaptive dual-layer sliding mode control (ADLSMC) [2] has been designed. This proposed controller can realize not only fixed-point tracking but also dynamic tracking, but the performance of the dual layer sliding mode control is very sensitive to the parameters. A variable gain super-twisting algorithm (VGSTA) and super-twisting-like algorithm (STLA) for the load frequency control of a two-area interconnected power system with nonlinearities was presented in [3]. This control strategy avoids the chattering phenomenon that may occur in traditional sliding mode control, but it is highly dependent on the system model. In [4], in order to handle the matched structured and unstructured uncertainties, a Lyapunov-based control concept was combined with a variable structure and adaptive control. It was demonstrated that the gains of the discontinuous control action may be remarkably reduced under the control scheme, while the stability analysis of the system becomes very complicated. An adaptive global prescribed performance tracking control (APPTC) scheme for nonlinear robotic systems in the presence of unknown control directions and nonparametric uncertainties has been developed [5]. This solution can ensure that the system meets the predefined performance criteria throughout its operation. Nevertheless, it requires the real-time execution of a large amount of computation, potentially impacting the timeliness of control and the system’s response speed. In [6], a novel adaptive backstepping nonsingular fast terminal sliding mode control (ABNFTSMC) was proposed to track the control of a PUMA560 robot. However, the major limitation of the proposed ABNFTSMC is that its design procedure depends on prior knowledge of the bound value of the disturbance and uncertainties. Due to the high nonlinearity, strong coupling, and time-varying characteristics of flexible joint robot manipulators, Ref. [7] used a neural network observer to solve the aforementioned problem. This controller is simple and straightforward, yet it requires complex parameter tuning and depends heavily on training data.

It is well known that robot manipulator systems typically suffer from disturbance and uncertainties. Therefore, the design of robust controllers has achieved great popularity. There is no doubt that the sliding mode controller (SMC) is the most promising robust controller. SMC provides robust and stable robot manipulator systems in the presence of disturbances and uncertainties and has also attracted a great deal of attention from many researchers [1,2,3,4,6,8,9,10,11]. Some papers have conducted in-depth work on the stabilization of uncertain robot manipulator systems, including SMC tracking control [2,5,6], adaptive SMC tracking control [8,12,13], SMC-based predictive control [9,14,15]. The SMCs used in this literature exhibit the chattering phenomenon. As this research was being developed, super-twisting algorithm (STA) came into being. STA [3,4] is a second-order sliding mode control technique designed to address the challenges of robust control in systems with uncertainties and disturbances. It is widely used because it provides finite-time convergence and robustness against disturbances without requiring explicit knowledge of the disturbance bounds. This effectively suppresses the chattering phenomenon in the system, enhancing its robustness and control accuracy.

In the above literature, the problems regarding practical applications have not been solved. However, digitization can be achieved by using complex digital computers, especially in practical robot manipulator systems. It is well known that the control signal is implemented in a digital periodic manner to realize time-triggered control [16,17,18,19,20]. That is to say, the evolution of the uncertain robot manipulator system state is independent after every τ period. This increases the usage of resources in robot systems when updating the state information after every τ period. In practical applications, designing a controller that saves computation is very important [19,20,21,22,23].

One promising control scheme, due to its simple control structure and robustness against robot system uncertainties, is event-triggered control [12,13,14,15,16,17,18,19,20,21,22,23,24]. This control scheme updates the state information only at discrete triggering instants, which leads to the non-periodic implementation of control. The triggering condition is the core of the event-triggered control, and the triggering instants are calculated using the triggering condition. In this technique, the computational cost and network bandwidth usage can be reduced. In [12,13,14], the authors designed an event-triggered controller and applied it to control a robot manipulator, achieving good control results. In [16], an event-triggering strategy is presented to deal with the stabilization of a linear time-invariant system. In [17,18], addressing the robust stabilization problem for a class of nonlinear systems subject to external disturbances, the authors used sliding mode control based on an event-triggering control scheme. In [24], the researchers presented an event-triggered-based sliding mode controller to track control for a class of uncertain Euler–Lagrange systems. In [19,20], an adaptive event-triggered sliding mode control scheme was designed for the attitude control of the Reusable Launch Vehicle in the presence of unknown external disturbances. However, as far as we know, there is relatively little research on how to use the event-triggered algorithm for uncertain robot manipulator systems.

Unlike the periodic sampling control algorithm [24], when the control input needs to be updated is determined by the event-triggering method. It is necessary for uncertain robot manipulator systems to save resources during the trajectory tracking performance. In addition, Zeno behavior often occurs in robot manipulator systems. This may lead to the control signal not being updated at the moment of triggering, which could lead to robot system instability [25,26]. Thus, Zeno behavior should be taken into account when designing the controller. The super-twisting algorithm showed good performance, with complete robustness of the robot system [27]. However, event-triggering based on super-twisting control schemes is rarely studied in the existing literature.

Motivated by the aforementioned discussions, the main contributions of this paper can be summarized as follows:

(i) To achieve the desired performance, an event-triggered control combined with the super-twisting technique is presented to handle uncertain robot manipulator systems in the presence of uncertainties and disturbances.

(ii) First, an adaptive event-triggered super-twisting controller is presented to deal with a class of multivariable nonlinear systems. The number of control updates can be reduced using the proposed scheme.

(iii) Second, the stability of a closed-loop system under the presented controller is analyzed using Lyapunov techniques.

The remainder of this article is organized as follows: Section 2 describes the robot manipulator systems and highlights the issue. The stability of the system and the ETASTC controller are rigorously outlined in Section 3. In Section 4, the proposed control scheme is applied to an uncertain manipulator system. The effectiveness of the proposed method is demonstrated through simulation in Section 5. Finally, Section 6 concludes this paper.

## 2. System Description and Problem Statement

The dynamic equation of robot manipulators can be mathematically formulated as [1,26,28]

(1) $$M\left(q\right)\ddot{q}+C\left(q,\dot{q}\right)\dot{q}+G\left(q\right)+F\left(\dot{q}\right)=\tau +\tau_{d}$$

where q, $\dot{q}$, and $\ddot{q}$ are the vectors of joint positions, velocities, and accelerations, respectively; $M\left(q\right)\in R^{n\times n}$ is the bounded positive-definite inertia matrix; $C\left(q,\dot{q}\right)\in R^{n\times n}$ is the centrifugal and Coriolis matrix; $G(q)\in R^{n}$ indicates the gravitational vector; $F\left(\dot{q}\right)\in R^{n}$ is the vector of viscous friction forces; τ is the control input torque; $\tau_{d}\in R^{n}$ denotes the external disturbances torque.

Designing a controller to track the desired trajectory in the presence of disturbances is the main objective of our research. Based on this, the desired trajectory for Equation (1) is defined as $x_{d}={\left[\begin{array}{cc} q_{d}^{T} & {\dot{q}}_{d}^{T} \end{array}\right]}^{T}$. $\xi_{1}$ and $\xi_{2}$ are denoted by $\xi_{1}=q-q_{d}$, $\xi_{2}=\dot{q}-{\dot{q}}_{d}$. Then, Equation (1) can be rewritten as

(2) $$\dot{\xi }=f\left(\xi ,x_{d}\right)+\psi_{1}\left(\xi ,x_{d}\right)\tau +B\left(\bar{d}-{\ddot{q}}_{d}\right)$$

where $\xi ={\left[\begin{array}{cc} \xi_{1}^{T} & \xi_{2}^{T} \end{array}\right]}^{T}$ is the tracking error and $f\left(\xi ,x_{d}\right)=\left[\begin{array}{c} \xi_{2}\\ g\left(\xi ,x_{d}\right) \end{array}\right]$, $\psi_{1}\left(\xi ,x_{d}\right)=\left[\begin{array}{c} 0\\ \psi \left(\xi ,x_{d}\right) \end{array}\right]$, $B=\left[\begin{array}{c} 0\\ I \end{array}\right]$ and $\bar{d}=\psi \left(\xi ,x_{d}\right)\tau_{d}$.

Here, $\psi \left(\xi ,x_{d}\right)=M^{-1}\left(\xi_{1}+q_{d}\right)$, and the vector $g\left(\xi ,x_{d}\right)$ is presented as

$$g\left(\xi ,x_{d}\right)=-\psi \left(\xi ,x_{d}\right)\left[\begin{array}{l} C\left(\xi_{1}+q_{d},\xi_{2}+{\dot{q}}_{d}\right)\left(\xi_{2}+{\dot{q}}_{d}\right)+\\ F\left(\xi_{2}+{\dot{q}}_{d}\right)+G\left(\xi_{1}+q_{d}\right) \end{array}\right]$$

From the above, we can see that the tracking problem in Equation (1) is simplified to the stabilization in Equation (2). Then, the control method can be presented to guarantee the stability of robot manipulator systems in the presence of uncertainties and disturbances.

## 3. Adaptive Super-Twisting Based on an Event-Triggered Scheme

The typical multivariable second-order system is given by the following formula [19]:

(3) $$\dot{x}=f\left(x\right)+b\left(u+d\right),x\left(0\right)=x\left(t_{0}\right)$$

where $x=\left[\begin{array}{cc} x_{1} & x_{2} \end{array}\right]\in R^{2n}$, $f\left(x\right)=\left[\begin{array}{cc} x_{2} & p\left(x\right) \end{array}\right]\in R^{2n}$, $p\left(x\right)\in R^{n}$ is the nonlinear function, x denotes the state vector, the value of vector b is $b=\left[\begin{array}{cc} 0 & 1 \end{array}\right]\in R^{2n\times n}$, $x_{1}$ and $x_{2}\in R^{n}$ are both state variables, $u\in R^{n}$ presents the control vector, and $d\in R^{n}$ is the disturbance vector. Additionally, the symbol $\left\|.\right\|$ represents the norm of a vector. We define the triggering instants as ${\left\{t_{k}\right\}}_{k=0}^{\infty }$ for any $k\in Z_{\geq 0}$ in the event-triggered control. Then, the discrete error between two continuous intervals is represented as $e\left(t_{k}\right)=x\left(t_{k}\right)-x\left(t\right)$ with $e\left(t_{k}\right)=x\left(t_{k}\right)-x\left(t_{k}\right)=0$, where $t\in \left[t_{k},t_{k+1}\right)$. For the subsequent research in this paper, we introduce the following assumptions.

**Assumption 1.** **([24]).**  *The function $f\left(x\right)$ in Equation (3) satisfies the Lipschitz property in a compact domain $\Phi \subset R^{2n}$, such that $\left\|f\left(z_{1}\right)-f\left(z_{2}\right)\right\|\leq K\left\|z_{1}-z_{2}\right\|$ for some Lipschitz constant K of function $f\left(x\right)$, respectively for the corresponding vector in Φ.*

**Assumption 2.** **([20]).**  *The disturbance $d\left(t\right)$ is assumed to be bounded, and there exist two bounded but unknown constants η and $\bar{\eta }$, such that $\left\|d\left(t\right)\right\|\leq \eta$ and $\left\|\dot{d}\left(t\right)\right\|\leq \bar{\eta }$.*

The sliding variable is designed as $s\left(t\right)=\mathrm{Nx}\left(t\right)=x_{1}\left(t\right)+x_{2}\left(t\right)$, with $N\in R^{2\times 4}$, and we define the sliding manifold as

(4) $$S=\left\{x\in R^{2n}:\left\|s\right\|=\left\|\mathrm{Nx}\right\|\leq \varepsilon \right\}$$

where ε>0. We call a control algorithm S the real (ideal) sliding mode surface function on the constraint μ=0 if it yields the real sliding mode surface for every initial condition in a finite time. It should be pointed out that the sliding manifold S is the real sliding surface [19,20]. Second, the triggering rules and control laws are designed to ensure the system trajectory reaches the sliding manifold. Then, the adaptive super-twisting controller based on the event-triggered scheme in Equation (3) is presented as

(5) $$u={\left(\mathrm{Nb}\right)}^{-1}\left[-\mathrm{Nf}\left(t_{k}\right)-\kappa \left(t_{k}\right){\left\|s\left(t_{k}\right)\right\|}^{\frac{1}{2}}signs\left(t_{k}\right)-\int_{t_{k}}^{t}\lambda \left(t_{k}\right)signs\left(t_{k}\right)dt\right]$$

For all $t\in \left[t_{k},t_{k+1}\right)$ and the adaptive gain κ, λ has upper bound $\left|\kappa \right|\leq \kappa^{*}$, $\left|\lambda \right|\leq \lambda^{*}$; then, κ and λ are designed as

(6) $$\begin{array}{l} \dot{\kappa }=rsign\left(\left\|s\right\|-\sigma \right)\\ \lambda =2w\kappa \end{array}$$

where r>0, w>0. Meanwhile, the adjustment parameter σ is selected as $\sigma =\alpha \left({\left\|s\left(t_{k}\right)\right\|}^{\frac{1}{2}}+\beta \right)/K,\;\alpha \in \left(0,1\right),\;\beta \in \left(0,\infty \right)$. Then, the triggering rule algorithm is designed as

(7) $$t_{k+1}=\inf\left\{t:t>t_{k},\;K\left\|N\right\|\left\|e\left(t\right)\right\|\geq \alpha \left({\left\|s\left(t_{k}\right)\right\|}^{\frac{1}{2}}+\beta \right)\right\}$$

This triggering scheme ensures

(8) $$K\left\|N\right\|\left\|e\left(t\right)\right\|<\alpha \left({\left\|s\left(t_{k}\right)\right\|}^{\frac{1}{2}}+\beta \right)$$

for all time t≥0.

**Theorem 1.**  *Considering the multivariable second-order system (3) with Assumptions 1 and 2, the sliding variable $s\left(t\right)$ will eventually reach the sliding band:*

(9) $$\Gamma =\left\{x\in {\mathbb{R}}^{2n}:\left\|s\left(t\right)\right\|=\left\|\mathrm{Nx}\left(t\right)\right\|\leq \max{\left(\frac{\Omega^{2}}{\left(\rho_{\min}\left(P\right)\partial \right)}\right)}^{2},\alpha \left({\left\|s\left(t_{k}\right)\right\|}^{\frac{1}{2}}+\beta \right)/K\right\}$$

*where the state variable $x\left(1\right)$ will converge to the band, as determined by the following formula:*

(10) $$\Lambda =\left\{x_{1}\in {\mathbb{R}}^{n}:\left\|x_{1}\right\|\leq \max{\left(\frac{\Omega^{2}}{\left(\rho_{\min}\left(P\right)\partial \right)}\right)}^{2},\alpha \left({\left\|s\left(t_{k}\right)\right\|}^{\frac{1}{2}}+\beta \right)/K\right\}$$

*with 0<∂<1; Ω is a positive constant given by (34), and P satisfies (36).*

**Proof of Theorem 1.**  Here, the proof can be divided into two steps: (i) if Equation (12), Equation (13), and Equation (14) are the triggering rule and control law conditions, the state variable s will converge to the sliding band (9); (ii) after the sliding trajectory enters the practical sliding manifold, we can rigorously prove the stability of the closed-loop system (3).

First step: First, using the control laws (5) and (6), and taking the derivative of $s\left(t\right)$, we can obtain the following:

(11) $$\begin{array}{ll} \dot{s}\left(t\right)= & \mathrm{Nf}\left(t\right)-\mathrm{Nf}\left(t_{k}\right)-\kappa \left(t_{k}\right){\left\|s\left(t_{k}\right)\right\|}^{\frac{1}{2}}signs\left(t_{k}\right)\\ & -\int_{t_{k}}^{t}\lambda \left(t_{k}\right)signs\left(t_{k}\right)dt+d\left(t\right) \end{array}$$

where $v\left(t\right)$ can be defined as $v\left(t\right)=-\int_{t_{k}}^{t}\lambda \left(t_{k}\right)signs\left(t_{k}\right)dt+d\left(t\right)$; then, Equation (11) is rearranged as

(12) $$\begin{array}{l} \left\{\begin{array}{l} \dot{s}\left(t\right)=\mathrm{Nf}\left(t\right)-\mathrm{Nf}\left(t_{k}\right)-\kappa \left(t_{k}\right){\left\|s\left(t_{k}\right)\right\|}^{\frac{1}{2}}signs\left(t_{k}\right)+v\left(t\right)\\ \dot{v}\left(t\right)=-\lambda \left(t_{k}\right)signs\left(t_{k}\right)+\dot{d}\left(t\right) \end{array}\right. \end{array}$$

For the purpose of analyzing the stability of Equation (12), we introduce a new notation as follows:

(13) $$\begin{array}{l} z\left(t\right)=\left[\begin{array}{c} z_{1}\left(t\right)\\ z_{2}\left(t\right) \end{array}\right]=\left[\begin{array}{c} {\left\|s\left(t\right)\right\|}^{\frac{1}{2}}signs\left(t\right)\\ v\left(t\right) \end{array}\right] \end{array}$$

After simple derivation, the time derivatives of $z_{1}\left(t\right)$ and $z_{2}\left(t\right)$ are

(14) $$\begin{array}{l} {\dot{z}}_{1}\left(t\right)=\frac{1}{2{\left\|s\left(t\right)\right\|}^{\frac{1}{2}}}\left[\mathrm{Nf}\left(t\right)-\mathrm{Nf}\left(t_{k}\right)-\kappa \left(t_{k}\right){\left\|s\left(t_{k}\right)\right\|}^{\frac{1}{2}}signs\left(t_{k}\right)+z_{2}\left(t\right)\right]\\ {\dot{z}}_{2}\left(t\right)=-\lambda \left(t_{k}\right)signs\left(t_{k}\right)+\dot{d}\left(t\right) \end{array}$$

Then, Equation (14) can be rearranged as

(15) $$\begin{array}{l} \dot{z}\left(t\right)=\frac{1}{\left\|z_{1}\left(t\right)\right\|}\left(l_{1}\left[\begin{array}{c} z_{1}\left(t_{k}\right)\\ z_{2}\left(t\right) \end{array}\right]+l_{2}\right) \end{array}$$

where

(16) $$\begin{array}{l} l_{1}=\left[\begin{array}{cc} -\frac{\kappa \left(t_{k}\right)}{2} & \frac{1}{2}\\ 0 & 0 \end{array}\right] \end{array}$$

and

(17) $$\begin{array}{l} l_{2}=\left[\begin{array}{c} \frac{1}{2}\mathrm{Nf}\left(t\right)-\frac{1}{2}\mathrm{Nf}\left(t_{k}\right)\\ -\lambda \left(t_{k}\right)\left\|z_{1}\left(t\right)\right\|signs\left(t_{k}\right)+\dot{d}\left(t\right)\left\|z_{1}\left(t\right)\right\| \end{array}\right] \end{array}$$

The Lyapunov candidate function is selected for Equation (14) as follows

(18) $$\begin{array}{l} V\left(z,\;\kappa ,\;\lambda \right)=V_{0}+\frac{1}{2}{\left(\kappa -\kappa^{*}\right)}^{2}+\frac{1}{2}{\left(\lambda -\lambda^{*}\right)}^{2} \end{array}$$

where

(19) $$\begin{array}{l} V_{0}=z{\left(t\right)}^{T}\mathrm{Qz}\left(t\right) \end{array}$$

with $Q=\left[\begin{array}{cc} \rho +4w^{2} & -2w\\ -2w & 1 \end{array}\right]$, ρ>0,w>0.

It should be pointed out that if ρ and w are both positive constants, we can conclude that the matrix Q is a positive definite symmetrical matrix. Considering $\left\|\dot{d}\left(t\right)\right\|\leq \bar{\eta }$, let us calculate the time derivative of $V_{0}$:

(20) $$\begin{array}{ll} {\dot{V}}_{0} & =\frac{1}{\left\|z_{1}\left(t\right)\right\|}\left\{z^{T}\left(t\right){\mathrm{Ql}}_{1}\left[\begin{array}{c} z_{1}\left(t_{k}\right)\\ z_{2}\left(t\right) \end{array}\right]+\left[\begin{array}{cc} z_{1}\left(t_{k}\right) & z_{2}\left(t\right) \end{array}\right]l_{1}^{T}\mathrm{Qz}\left(t\right)+2z^{T}\left(t\right){\mathrm{Ql}}_{2}\right\}\\ & \leq \frac{1}{\left\|z_{1}\left(t\right)\right\|}\left\{\begin{array}{l} -\left(\rho +4w^{2}\right)\kappa \left(t_{k}\right)z_{1}^{T}\left(t\right)z_{1}\left(t_{k}\right)+2w\kappa \left(t_{k}\right)z_{2}^{T}\left(t\right)z_{1}\left(t_{k}\right)\\ +\left(\rho +4w^{2}\right)z_{2}^{T}\left(t\right)z_{1}\left(t\right)+\left[2w\lambda \left(t_{k}\right)+2w\bar{\eta }\right]z_{1}^{T}\left(t\right)z_{1}\left(t\right)\\ -2wz_{2}^{T}\left(t\right)z_{2}\left(t\right)-\lambda \left(t_{k}\right)z_{2}^{T}\left(t\right)\left\|z_{1}\left(t\right)\right\|signs\left(t_{k}\right)\\ +\bar{\eta }z_{2}^{T}\left(t\right)z_{1}\left(t\right)+\left[\left(\rho +4w^{2}\right)/2\right]z_{1}^{T}\left(t\right)N\left[f\left(t\right)-f\left(t_{k}\right)\right]\\ -wz_{2}^{T}\left(t\right)N\left[f\left(t\right)-f\left(t_{k}\right)\right] \end{array}\right\} \end{array}$$

It is worth noting that two different situations should be discussed. One situation is that $signs\left(t_{k}\right)=signs\left(t\right)$. The other situation is $signs\left(t_{k}\right)\neq signs\left(t\right)$. Then, we can prove situations 1 and 2, respectively.

Case 1: Assuming that $signs\left(t_{k}\right)=signs\left(t\right)$, we can easily obtain $signs\left(t_{k}\right)=signs\left(t\right)$ and $\left\|s\left(t_{k}\right)\right\|>\sigma$, $\dot{\kappa }>0$. Based on Assumption 1 and $z_{1}^{T}z_{2}=z_{2}^{T}z_{1}\leq \left\|z_{1}\right\|\left\|z_{2}\right\|$, $\left\|f\left(t\right)-f\left(t_{k}\right)\right\|\leq K\left\|x\left(t\right)-x\left(t_{k}\right)\right\|\leq K\left\|e\left(t\right)\right\|$, Equation (20) can be rewritten as

(21) $$\begin{array}{ll} {\dot{V}}_{0} & \leq \frac{1}{\left\|z_{1}\left(t\right)\right\|}\left\{\begin{array}{l} \left[2w\lambda \left(t_{k}\right)+2w\bar{\eta }\right]z_{1}^{T}\left(t\right)z_{1}\left(t\right)-2wz_{2}^{T}\left(t\right)z_{2}\left(t\right)\\ +\left[\rho +4w^{2}-\lambda \left(t_{k}\right)+\bar{\eta }\right]z_{2}^{T}\left(t\right)z_{1}\left(t\right)-\left(\rho +4w^{2}\right)\kappa \left(t_{k}\right)z_{1}^{T}\left(t\right)z_{1}\left(t_{k}\right)\\ +2w\kappa \left(t_{k}\right)z_{2}^{T}\left(t\right)z_{2}\left(t_{k}\right)+\frac{\left(\rho +4w^{2}\right)}{2}z_{1}^{T}\left(t\right)N\left[f\left(t\right)-f\left(t_{k}\right)\right]\\ -wz_{2}^{T}\left(t\right)N\left[f\left(t\right)-f\left(t_{k}\right)\right] \end{array}\right\}\\ & \leq \frac{1}{\left\|z_{1}\left(t\right)\right\|}\left\{\begin{array}{l} \left[2w\lambda \left(t_{k}\right)+2w\bar{\eta }\right]z_{1}^{T}\left(t\right)z_{1}\left(t\right)-2wz_{2}^{T}\left(t\right)z_{2}\left(t\right)\\ +\left[\rho +4w^{2}-\lambda \left(t_{k}\right)+\bar{\eta }\right]z_{2}^{T}\left(t\right)z_{1}\left(t\right)-\left(\rho +4w^{2}\right)\kappa \left(t_{k}\right)\left\|z_{1}\left(t\right)\right\|\left\|z_{1}\left(t_{k}\right)\right\|\\ +2w\kappa \left(t_{k}\right)\left\|z_{2}\left(t\right)\right\|\left\|z_{1}\left(t_{k}\right)\right\|+\frac{\left(\rho +4w^{2}\right)}{2}\left\|z_{1}\left(t\right)\right\|KN\left\|e\left(t\right)\right\|\\ +w\left\|z_{2}\left(t\right)\right\|KN\left\|e\left(t\right)\right\| \end{array}\right\} \end{array}$$

Due to $\left\|s\left(t_{k}\right)\right\|=\left\|s\left(t\right)+N\left[x\left(t_{k}\right)-x\left(t\right)\right]\right\|$, the following inequality can be obtained:

(22) $$\begin{array}{l} \left\|s\left(t\right)\right\|-\left\|N\right\|\left\|e\left(t\right)\right\|\leq \left\|s\left(t_{k}\right)\right\|\leq \left\|s\left(t\right)\right\|+\left\|N\right\|\left\|e\left(t\right)\right\| \end{array}$$

From Equation (8) and Equation (22), we can obtain

(23) $$\begin{array}{l} \left\|s\left(t\right)\right\|-\frac{{\left\|s\left(t_{k}\right)\right\|}^{\frac{1}{2}}+\beta }{K}\leq \left\|s\left(t_{k}\right)\right\|\leq \left\|s\left(t\right)\right\|+\frac{{\left\|s\left(t_{k}\right)\right\|}^{\frac{1}{2}}+\beta }{K} \end{array}$$

After a simple derivation of the right side of inequality (23), we can obtain

(24) $$\begin{array}{l} {\left({\left\|s\left(t_{k}\right)\right\|}^{\frac{1}{2}}-\frac{1}{2K}\right)}^{2}\leq \left\|s\left(t\right)\right\|+\frac{1}{4K^{2}}+\frac{\beta }{K} \end{array}$$

Taking the square root of both sides of Equation (24), it follows that

(25) $$\begin{array}{l} \left|{\left\|s\left(t_{k}\right)\right\|}^{\frac{1}{2}}-\frac{1}{2K}\right|\leq {\left(\left\|s\left(t\right)\right\|+\frac{1}{4K^{2}}+\frac{\beta }{K}\right)}^{\frac{1}{2}} \end{array}$$

According to the inequality ${\left(a+b\right)}^{\frac{1}{2}}\leq a^{\frac{1}{2}}+b^{\frac{1}{2}}$, which holds for any a>0 and b>0, the above inequality (25) can be given by the following:

(26) $$\begin{array}{l} {\left\|s\left(t_{k}\right)\right\|}^{\frac{1}{2}}\leq {\left\|s\left(t\right)\right\|}^{\frac{1}{2}}+\frac{1}{2K}+{\left(\frac{1}{4K^{2}}+\frac{\beta }{K}\right)}^{\frac{1}{2}} \end{array}$$

Furthermore, by calculating the left side of inequality (22), we can obtain

(27) $$\begin{array}{l} {\left({\left\|s\left(t_{k}\right)\right\|}^{\frac{1}{2}}+\frac{1}{2K}\right)}^{2}+\frac{\beta }{K}\geq \left\|s\left(t\right)\right\|+\frac{1}{4K^{2}}\geq \left\|s\left(t\right)\right\| \end{array}$$

Then, according to the inequality ${\left(a+b\right)}^{\frac{1}{2}}\leq a^{\frac{1}{2}}+b^{\frac{1}{2}}$, Equation (27) can be rearranged as

(28) $$\begin{array}{l} {\left\|s\left(t_{k}\right)\right\|}^{\frac{1}{2}}\geq {\left\|s\left(t\right)\right\|}^{\frac{1}{2}}-\left(\frac{1}{2K}+{\left(\frac{\beta }{K}\right)}^{\frac{1}{2}}\right) \end{array}$$

Finally, combining inequalities (26) and (28), we can obtain

(29) $$\begin{array}{l} {\left\|s\left(t\right)\right\|}^{\frac{1}{2}}-\left(\frac{1}{2K}+{\left(\frac{\beta }{K}\right)}^{\frac{1}{2}}\right)\leq {\left\|s\left(t_{k}\right)\right\|}^{\frac{1}{2}}\leq {\left\|s\left(t\right)\right\|}^{\frac{1}{2}}+\frac{1}{2K}+{\left(\frac{1}{4K^{2}}+\frac{\beta }{K}\right)}^{\frac{1}{2}} \end{array}$$

According to definition (13), this leads to

(30) $$\begin{array}{l} \left\|z_{1}\left(t\right)\right\|-\left(\frac{1}{2K}+{\left(\frac{\beta }{K}\right)}^{\frac{1}{2}}\right)\leq \left\|z_{1}\left(t_{k}\right)\right\|\leq \left\|z_{1}\left(t\right)\right\|+\frac{1}{2K}+{\left(\frac{1}{4K^{2}}+\frac{\beta }{K}\right)}^{\frac{1}{2}} \end{array}$$

Substituting inequality (30) into inequality (21), it follows that

(31) $$\begin{array}{ll} {\dot{V}}_{0} & \leq \frac{1}{\left\|z_{1}\left(t\right)\right\|}\left\{\begin{array}{l} \left[2w\lambda \left(t_{k}\right)+2w\bar{\eta }\right]z_{1}^{T}\left(t\right)z_{1}\left(t\right)-2wz_{2}^{T}\left(t\right)z_{2}\left(t\right)\\ +\left[\rho +4w^{2}-\lambda \left(t_{k}\right)+\bar{\eta }\right]z_{2}^{T}\left(t\right)z_{1}\left(t\right)-\left(\rho +4w^{2}\right)\kappa \left(t_{k}\right)\left\|z_{1}\left(t\right)\right\|\left(\left\|z_{1}\left(t\right)\right\|-\left(\frac{1}{2K}+{\left(\frac{\beta }{K}\right)}^{\frac{1}{2}}\right)\right)\\ +2w\kappa \left(t_{k}\right)\left\|z_{2}\left(t\right)\right\|\left(\left\|z_{1}\left(t\right)\right\|+\frac{1}{2K}+{\left(\frac{1}{4K^{2}}+\frac{\beta }{K}\right)}^{\frac{1}{2}}\right)+w\left\|z_{2}\left(t\right)\right\|\left(\left\|z_{1}\left(t_{k}\right)\right\|+\beta \right)\\ +\frac{\left(\rho +4w^{2}\right)}{2}\left\|z_{1}\left(t\right)\right\|\left(\left\|z_{1}\left(t_{k}\right)\right\|+\beta \right) \end{array}\right\}\\ & \leq \frac{1}{\left\|z_{1}\left(t\right)\right\|}\left\{\begin{array}{l} \left[2w\lambda \left(t_{k}\right)+2w\bar{\eta }\right]z_{1}^{T}\left(t\right)z_{1}\left(t\right)-2wz_{2}^{T}\left(t\right)z_{2}\left(t\right)\\ +\left[\rho +4w^{2}-\lambda \left(t_{k}\right)+\bar{\eta }\right]z_{2}^{T}\left(t\right)z_{1}\left(t\right)-\left(\rho +4w^{2}\right)\kappa \left(t_{k}\right)\left\|z_{1}\left(t\right)\right\|\left(\left\|z_{1}\left(t\right)\right\|-\left(\frac{1}{2K}+{\left(\frac{\beta }{K}\right)}^{\frac{1}{2}}\right)\right)\\ +2w\kappa \left(t_{k}\right)\left\|z_{2}\left(t\right)\right\|\left(\left\|z_{1}\left(t\right)\right\|+\frac{1}{2K}+{\left(\frac{1}{4K^{2}}+\frac{\beta }{K}\right)}^{\frac{1}{2}}\right)+w\left\|z_{2}\left(t\right)\right\|\left(\left\|z_{1}\left(t\right)\right\|+\frac{1}{2K}+{\left(\frac{1}{4K^{2}}+\frac{\beta }{K}\right)}^{\frac{1}{2}}+\beta \right)\\ +\frac{\left(\rho +4w^{2}\right)}{2}\left\|z_{1}\left(t\right)\right\|\left(\left\|z_{1}\left(t\right)\right\|+\frac{1}{2K}+{\left(\frac{1}{4K^{2}}+\frac{\beta }{K}\right)}^{\frac{1}{2}}+\beta \right) \end{array}\right\} \end{array}$$

After a simple calculation, Equation (31) can be given by

(32) $$\begin{array}{l} {\dot{V}}_{0}\leq \frac{1}{\left\|z_{1}\left(t\right)\right\|}\left\{\begin{array}{l} \left[-\left(\rho +4w^{2}\right)\kappa \left(t_{k}\right)+2w\lambda \left(t_{k}\right)+2w\bar{\eta }+\frac{\left(\rho +4w^{2}\right)}{2}\right].{\left\|z_{1}\left(t\right)\right\|}^{2}\\ +\left[\rho +4w^{2}-\lambda \left(t_{k}\right)+\bar{\eta }+2w\kappa \left(t_{k}\right)+w\right].\left\|z_{2}\left(t\right)\right\|\left\|z_{1}\left(t\right)\right\|-2w{\left\|z_{2}\left(t\right)\right\|}^{2} \end{array}\right\}\\ \;\;\;\;+V_{1} \end{array}$$

where

(33) $$\begin{array}{ll} V_{1} & \leq \frac{1}{\left\|z_{1}\left(t\right)\right\|}\left\{\begin{array}{l} \left[\left(\rho +4w^{2}\right)\kappa \left(t_{k}\right)\left(\frac{1}{2K}+{\left(\frac{\beta }{K}\right)}^{\frac{1}{2}}\right)+\frac{\left(\rho +4w^{2}\right)}{2}\left(\frac{1}{2K}+{\left(\frac{1}{4K^{2}}+\frac{\beta }{K}\right)}^{\frac{1}{2}}+\beta \right)\right]\left\|z_{1}\left(t\right)\right\|\\ +\left[2w\kappa \left(t_{k}\right)\left(\frac{1}{2K}+{\left(\frac{1}{4K^{2}}+\frac{\beta }{K}\right)}^{\frac{1}{2}}\right)+w\left(\frac{1}{2K}+{\left(\frac{1}{4K^{2}}+\frac{\beta }{K}\right)}^{\frac{1}{2}}+\beta \right)\right]\left\|z_{2}\left(t\right)\right\| \end{array}\right\}\\ & \leq \frac{1}{\left\|z_{1}\left(t\right)\right\|}\left\{\begin{array}{l} \left(\rho +4w^{2}\right)\left[\kappa^{*}\left(\frac{1}{2K}+{\left(\frac{\beta }{K}\right)}^{\frac{1}{2}}\right)+\frac{1}{2}\left(\frac{1}{2K}+{\left(\frac{1}{4K^{2}}+\frac{\beta }{K}\right)}^{\frac{1}{2}}\right)+\frac{1}{2}\beta \right]\\ +w\left[2\kappa^{*}\left(\frac{1}{2K}+{\left(\frac{1}{4K^{2}}+\frac{\beta }{K}\right)}^{\frac{1}{2}}\right)+\left(\frac{1}{2K}+{\left(\frac{1}{4K^{2}}+\frac{\beta }{K}\right)}^{\frac{1}{2}}\right)+\beta \right] \end{array}\right\}\left\|z\left(t\right)\right\|\\ & =\frac{\Omega }{\left\|z_{1}\left(t\right)\right\|}\left\|z\left(t\right)\right\| \end{array}$$

in which

(34) $$\begin{array}{ll} \Omega & =\left(\rho +4w^{2}\right)\left[\kappa^{*}\left(\frac{1}{2K}+{\left(\frac{\beta }{K}\right)}^{\frac{1}{2}}\right)+\frac{1}{2}\left(\frac{1}{2K}+{\left(\frac{1}{4K^{2}}+\frac{\beta }{K}\right)}^{\frac{1}{2}}\right)+\frac{1}{2}\beta \right]\\ & +w\left[2\kappa^{*}\left(\frac{1}{2K}+{\left(\frac{1}{4K^{2}}+\frac{\beta }{K}\right)}^{\frac{1}{2}}\right)+\left(\frac{1}{2K}+{\left(\frac{1}{4K^{2}}+\frac{\beta }{K}\right)}^{\frac{1}{2}}\right)+\beta \right] \end{array}$$

According to (34), Ω is bounded and Ω>0. Next, Equation (31) can be rearranged as follows:

(35) $$\begin{array}{ll} {\dot{V}}_{0} & \leq \frac{-1}{\left\|z_{1}\left(t\right)\right\|}\left[\begin{array}{cc} \left\|z_{1}\left(t\right)\right\| & \left\|z_{2}\left(t\right)\right\| \end{array}\right]P\left[\begin{array}{c} \left\|z_{1}\left(t\right)\right\|\\ \left\|z_{2}\left(t\right)\right\| \end{array}\right]+V_{1}\\ & \leq \frac{-1}{\left\|z_{1}\left(t\right)\right\|}\chi^{T}\left(t\right)P\chi \left(t\right)+\frac{\Omega }{\left\|z_{1}\left(t\right)\right\|}\left\|z\left(t\right)\right\| \end{array}$$

where $\chi \left(t\right)={\left[\begin{array}{cc} z_{1}\left(t\right) & z_{2}\left(t\right) \end{array}\right]}^{T}$ and the matrix P are shown by

(36) $$ccP=\left[\begin{array}{cc} \left(\rho +4w^{2}\right)\kappa \left(t_{k}\right)-2w\lambda \left(t_{k}\right)-2w\bar{\eta }-\frac{\left(\rho +4w^{2}\right)}{2} & *\\ -\frac{1}{2}\left(\rho +4w^{2}-\lambda \left(t_{k}\right)+\bar{\eta }+2w\kappa \left(t_{k}\right)+w\right) & 2w \end{array}\right]$$

We set λ=2wκ to make sure P is a positive definite matrix. The matrix P is positive at the minimal eigenvalue $\rho_{\min}\left(P\right)\geq w$ if

(37) $$\kappa \left(t\right)\geq \kappa \left(t_{k}\right)>\frac{\rho +4w^{2}+2w\left(1+\bar{\eta }\right)}{8\rho }+\frac{{\left(\rho +4w^{2}+\bar{\eta }+w\right)}^{2}}{4\rho w}$$

Then, considering the positive definiteness of P and $\left\|\chi \right\|=\left\|z\right\|$, the following can be obtained:

(38) $$\rho_{\min}\left(P\right){\left\|z\right\|}^{2}\leq \chi^{T}P\chi \leq \rho_{\max}\left(P\right){\left\|z\right\|}^{2}$$

where $\rho_{\min}\left(P\right)$ is the minimum eigenvalue of the matrix P, and $\rho_{\max}\left(P\right)$ is the maximum eigenvalue of the matrix P. Considering $\left\|z_{1}\right\|\leq \left\|z\right\|$ and Equation (38), ${\dot{V}}_{0}$ in (35) becomes

(39) $${\dot{V}}_{0}\leq -\frac{1}{\left\|z_{1}\left(t\right)\right\|}\rho_{\min}\left(P\right){\left\|z\left(t\right)\right\|}^{2}+\frac{\Omega }{\left\|z_{1}\left(t\right)\right\|}\left\|z\left(t\right)\right\|$$

Based on (39) and $\dot{\kappa }\geq 0$, taking the derivative of V with respect to time in Equation (18) satisfies the following inequality:

(40) $$\begin{array}{ll} \dot{V} & \leq -\frac{1}{\left\|z_{1}\left(t\right)\right\|}\rho_{\min}\left(P\right){\left\|z\left(t\right)\right\|}^{2}+\frac{\Omega }{\left\|z_{1}\left(t\right)\right\|}\left\|z\left(t\right)\right\|\\ & \;\;+\left(\kappa -\kappa^{*}\right)\dot{\kappa }+\left(\lambda -\lambda^{*}\right)\dot{\lambda }\\ & =-\frac{1}{\left\|z_{1}\left(t\right)\right\|}\rho_{\min}\left(P\right){\left\|z\left(t\right)\right\|}^{2}+\frac{\Omega }{\left\|z_{1}\left(t\right)\right\|}\left\|z\left(t\right)\right\|\\ & \;\;-\left|\kappa -\kappa^{*}\right|r-\left|\lambda -\lambda^{*}\right|2wr\\ & \leq -\frac{1}{\left\|z_{1}\left(t\right)\right\|}\rho_{\min}\left(P\right)\left(1-\partial \right){\left\|z\left(t\right)\right\|}^{2}-\left|\kappa -\kappa^{*}\right|r\\ & \;\;-\left|\lambda -\lambda^{*}\right|2wr-\frac{1}{\left\|z_{1}\left(t\right)\right\|}\rho_{\min}\left(P\right)\partial {\left\|z\left(t\right)\right\|}^{2}+\frac{\Omega }{\left\|z_{1}\left(t\right)\right\|}\left\|z\left(t\right)\right\| \end{array}$$

where 0<∂<1. According to Equation (19), ${\left(V_{0}/\rho_{\max}\left(Q\right)\right)}^{\frac{1}{2}}\leq \left\|z\right\|\leq {\left(V_{0}/\rho_{\min}\left(Q\right)\right)}^{\frac{1}{2}}$, Equation (40) can become

(41) $$\begin{array}{ll} \dot{V} & \leq -\gamma V_{0}^{\frac{1}{2}}-\left|\kappa -\kappa^{*}\right|r-\left|\lambda -\lambda^{*}\right|2wr\\ & -\frac{1}{\left\|z_{1}\left(t\right)\right\|}\rho_{\min}\left(P\right)\partial {\left\|z\left(t\right)\right\|}^{2}+\frac{\Omega }{\left\|z_{1}\left(t\right)\right\|}\left\|z\left(t\right)\right\| \end{array}$$

where $\gamma =\rho_{\min}\left(P\right)\left(1-\partial \right)/\sqrt{\rho_{\min}\left(Q\right)}$. Taking into account the inequality ${\left(a^{2}+b^{2}+c^{2}\right)}^{\frac{1}{2}}\leq \left|a\right|+\left|b\right|+\left|c\right|$, Equation (41) can be given by

(42) $$\dot{V}\leq -\gamma V_{0}^{\frac{1}{2}}-\frac{1}{\left\|z_{1}\left(t\right)\right\|}\rho_{\min}\left(P\right)\partial {\left\|z\left(t\right)\right\|}^{2}+\frac{\Omega }{\left\|z_{1}\left(t\right)\right\|}\left\|z\left(t\right)\right\|$$

where $\gamma_{0}=\min\left(\gamma ,\sqrt{2}r,2\sqrt{2}wr\right)$. According to Equation (42), it can be seen that $\dot{V}\leq -\gamma_{0}V^{\frac{1}{2}}$ if $\left\|z_{1}\left(t\right)\right\|>\Omega /\left(\rho_{\min}\left(P\right)\partial \right)$. From [20], system (14) is stable, and the state variable will ultimately converge to region $\left\{z\left(t\right)\in {\mathbb{R}}^{n}:\left\|z\left(t\right)\right\|\leq \Omega /\left(\rho_{\min}\left(P\right)\partial \right)\right\}$ in a finite time. Based on the inequality $\left\|z_{1}\right\|\leq \left\|z\right\|$ and Equation (13), the sliding variable converges to the following band:

(43) $$\left\|s\left(t\right)\right\|\leq \frac{\Omega^{2}}{{\left(\rho_{\min}\left(P\right)\partial \right)}^{2}}$$

Case 2: It can be seen from relation (7) that an increase in the sliding trajectory to exceed a certain value is not allowed. Here, when $signs\left(t_{k}\right)\neq signs\left(t\right)$, the sliding trajectory is bounded at last. Find the maximum deviation of the sliding trajectory in any time interval $\left[\begin{array}{cc} t_{k} & t_{k+1} \end{array}\right)$, and the limit bound can be calculated using the following formula:

(44) $$\begin{array}{ll} \left\|s\left(t_{k}\right)-s\left(t\right)\right\| & =\left\|\mathrm{Nx}\left(t_{k}\right)-\mathrm{Nx}\left(t\right)\right\|\\ & \leq \left\|N\right\|\left\|e\left(t\right)\right\|\\ & \leq \frac{\alpha \left({\left\|s\left(t_{k}\right)\right\|}^{\frac{1}{2}}+\beta \right)}{K} \end{array}$$

If $s\left(t_{k}\right)=0$, the maximum value of $s\left(t\right)$ is given by

(45) $$\left\{x\in {\mathbb{R}}^{2n}:\left\|s\left(t\right)\right\|\leq \frac{\alpha \left({\left\|s\left(t_{k}\right)\right\|}^{\frac{1}{2}}+\beta \right)}{K}\right\}$$

Through the above analysis, the sliding variable $s\left(t\right)$ ultimately enters the region as follows:

(46) $$\left\|s\left(t\right)\right\|\leq \max\left(\frac{\Omega^{2}}{{\left(\rho_{\min}\left(P\right)\partial \right)}^{2}},\frac{\alpha \left({\left\|s\left(t_{k}\right)\right\|}^{\frac{1}{2}}+\beta \right)}{K}\right)$$

and remains there.

Next, we prove that the state of the closed-loop system (3) also converges to the bounded (10) when the sliding variable enters the region Γ. We design the Lyapunov function as $V_{2}=x_{1}^{T}x_{1}$; taking the derivative of $V_{2}$ along the system trajectory of Equation (3), one can obtain the following:

(47) $$\begin{array}{ll} {\dot{V}}_{2} & ={\dot{x}}_{1}^{T}x_{1}+x_{1}^{T}{\dot{x}}_{1}=2x_{1}^{T}x_{2}\\ & \leq 2x_{1}^{T}\left(-x_{1}+s\right)\\ & \leq -2{\left\|x_{1}\right\|}^{2}+2\left\|x_{1}\right\|\left\|s\left(t\right)\right\| \end{array}$$

Considering Equation (46), vector $x_{1}\left(t\right)$ is eventually bounded in region Λ.

(48) $$\left\|x_{1}\left(t\right)\right\|\leq \max\left(\frac{\Omega^{2}}{{\left(\rho_{\min}\left(P\right)\partial \right)}^{2}},\frac{\alpha \left({\left\|s\left(t_{k}\right)\right\|}^{\frac{1}{2}}+\beta \right)}{K}\right)$$

Thus, the proof is completed. □

Next, we discuss how the triggering sequences of the ETASTC must be avoided for Zeno execution. The event interval time $T_{k}:=t_{k+1}-t_{k}$ for any $k\in R_{\geq 0}$ is introduced. Our main result is provided by the following important theorem.

**Theorem 2.**  *Considering the closed-loop system (3), the triggering sequences are admissible and Zeno free, in which the $T_{k}$ lower bound is always positive.*

**Proof of Theorem 2.**  Taking into account $Τ=\left\{t\in \left[t_{k},\infty \right):\left\|e\left(t\right)\right\|=0\right\}$, for all $t\in \left[t_{k},\infty \right)\backslash Τ$, one can obtain

(49) $$\begin{array}{ll} \frac{d}{dt}\left\|e\left(t\right)\right\| & \leq \left\|\frac{de\left(t\right)}{dt}\right\|=\left\|\frac{dx\left(t\right)}{dt}\right\|\\ & =\left\|\begin{array}{l} f\left(t\right)-f\left(t_{k}\right)-N^{-1}\kappa \left(t_{k}\right){\left\|s\left(t_{k}\right)\right\|}^{\frac{1}{2}}signs\left(t_{k}\right)\\ -N^{-1}\lambda \left(t_{k}\right)\int_{t_{k}}^{t}signs\left(t_{k}\right)dt+\mathrm{bd}\left(t\right) \end{array}\right\|\\ & \leq \left\|f\left(t\right)-f\left(t_{k}\right)\right\|+\left\|N^{-1}\right\|\left|\kappa \left(t_{k}\right)\right|{\left\|s\left(t_{k}\right)\right\|}^{\frac{1}{2}}\\ & \;\;+\left\|N^{-1}\right\|\left|\lambda \left(t_{k}\right)\right|\left(t-t_{k}\right)+\left\|b\right\|\left\|d\left(t\right)\right\| \end{array}$$

Based on assumptions 1 and 2, (49) can be rewritten as

(50) $$\frac{d}{dt}\left\|e\left(t\right)\right\|\leq K\left\|e\left(t\right)\right\|+h+\left\|N^{-1}\right\|\left|\lambda \left(t_{k}\right)\right|T_{k}$$

where $h=\left|\kappa \left(t_{k}\right)\right|\left\|N^{-1}\right\|{\left\|s\left(t_{k}\right)\right\|}^{\frac{1}{2}}+\left\|b\right\|\eta$ and the solution of differential inequality (50) can be calculated using the following formula:

(51) $$\left\|e\left(t\right)\right\|\leq \frac{l+\left\|N^{-1}\right\|\left|\lambda \left(t_{k}\right)\right|T_{k}}{K}\left(e^{K\left(t-t_{k}\right)}-1\right)$$

for $t\in \left[t_{k},t_{k+1}\right)$. As $\left\|e\left(t\right)\right\|$ increases to $\max\left\{{\left(\Omega^{2}/\left(\rho_{\min}\left(P\right)\partial \right)\right)}^{2},\alpha \left({\left\|s\left(t_{k}\right)\right\|}^{\frac{1}{2}}+\beta \right)/K\right\}$ in the interval $\left[t_{k},t_{k+1}\right)$, we can obtain

(52) $$X\leq \frac{l+\left\|N^{-1}\right\|\left|\lambda \left(t_{k}\right)\right|T_{k}}{K}\left(e^{K\left(t-t_{k}\right)}-1\right)$$

where $X=\max\left\{{\left(\Omega^{2}/\left(\rho_{\min}\left(P\right)\partial \right)\right)}^{2},\alpha \left({\left\|s\left(t_{k}\right)\right\|}^{\frac{1}{2}}+\beta \right)/K\right\}$; then, we define

(53) $$\Pi \left(T_{k}\right)=:\frac{l+\left\|N^{-1}\right\|\left|\lambda \left(t_{k}\right)\right|T_{k}}{K}\left(e^{KT_{k}}-1\right)-X$$

It should be pointed out that for arbitrary $T_{k}>0$ and $\Pi \left(0\right)=-X<0$, the $\Pi \left(T_{k}\right)$ is a monotonically increasing function. Then, we can obtain the solution of the inequality (53) as $T_{k}=X>0$, and $\Pi \left(X\right)=0$. It is easy to see that the $T_{k}$ lower bound is always positive for all $x\in R^{2n}$.

(54) $$T_{k}>0$$

From the above proof, we can see that the Zeno behavior of the control scheme does not exist, and the proof is complete. □

## 4. Application in Uncertain Robot Manipulators

In this section, according to (2), the error dynamics of the uncertain robot manipulators can be calculated as

(55) $$\begin{array}{l} {\dot{\xi }}_{1}{=\xi }_{2}\\ {\dot{\xi }}_{2}=\underset{F^{\prime }}{\underset{︸}{\left(-M^{-1}\left[C\left(\xi_{1}+q_{d},\xi_{2}+{\dot{q}}_{d}\right)\left(\xi_{2}+{\dot{q}}_{d}\right)+F\left(\xi_{2}+{\dot{q}}_{d}\right)+G\left(\xi_{1}+q_{d}\right)\right]\right)-{\;\ddot{q}}_{d}}}+\underset{\tau^{\prime }}{\underset{︸}{M^{-1}\tau }}+\underset{D^{\prime }\left(t\right)}{\underset{︸}{M^{-1}\tau_{d}}} \end{array}$$

The error dynamic of (55) can be rewritten as

(56) $$\dot{E}=F^{\prime }\left(E\right)+B\left(\tau^{\prime }+D^{\prime }\left(t\right)\right)$$

where $\dot{E}=\left[\begin{array}{cc} \xi_{1} & \xi_{2} \end{array}\right]\in R^{4}$, $F^{\prime }=\left[\begin{array}{cc} \xi_{2} & F \end{array}\right]\in R^{4}$, and $B=\left[\begin{array}{cc} 0 & 1 \end{array}\right]\in R^{4\times 2}$. $F^{\prime }\left(E\right)$ and $D^{\prime }\left(t\right)$ are given by the following assumptions.

**Assumption 3.**  *The function $F^{\prime }\left(\cdot \right)$ satisfies the Lipschitz condition in a compact domain $\Phi^{\prime }\subset R^{2}$ with a Lipschitz constant $K_{1}$, and can be written as $\left\|F^{\prime }\left(y_{1}\right)-F^{\prime }\left(y_{2}\right)\right\|\leq K_{1}\left\|y_{1}-y_{2}\right\|$ for the vectors $y_{1}$ and $y_{2}$ in $\Phi^{\prime }$.*

**Assumption 4.**  *The uncertain disturbance $D^{\prime }\left(t\right)$ in (56) is to be bounded; simultaneously, the inequalities $\left\|D^{\prime }\left(t\right)\right\|\leq \eta^{\prime }$ and $\left\|{\dot{D}}^{\prime }\left(t\right)\right\|\leq {\bar{\eta }}^{\prime }$ hold, in which $\eta^{\prime }$ and ${\bar{\eta }}^{\prime }$ are unknown as bounded constants.*

Next, the ETASTC algorithm is applied in an uncertain robot manipulator system with assumptions 3 and 4. Then, the sliding mode variable is given by the following formula:

(57) $$s^{\prime }=N^{\prime }E=\xi_{1}+\xi_{2}$$

where $N^{\prime }=\left[\begin{array}{cc} 1 & 1 \end{array}\right]\in R^{2\times 4}$ and the actual effective sliding manifold is given by $S^{\prime }=\left\{E\in {\mathbb{R}}^{4}\left|s^{\prime }=N^{\prime }E\leq \varepsilon^{\prime }\right.\right\}$, with $\varepsilon^{\prime }>0$. Then, the adaptive super-twisting controller based on the event-triggering algorithm is presented as

(58) $$u^{\prime }={\left(N^{\prime }B\right)}^{-1}\left[-N^{\prime }F^{\prime }\left(E\left(t_{k}\right)\right)-\kappa^{\prime }\left(t_{k}\right){\left\|s^{\prime }\left(t_{k}\right)\right\|}^{\frac{1}{2}}signs^{\prime }\left(t_{k}\right)-\int_{t_{k}}^{t}\lambda^{\prime }\left(t_{k}\right)signs^{\prime }\left(t_{k}\right)dt\right]$$

for all $t\in \left[t_{k},t_{k+1}\right)$, and the adaptive gain parameters $\kappa^{\prime }$, $\lambda^{\prime }$ are given by

(59) $$\left\{\begin{array}{l} {\dot{\kappa }}^{\prime }=rsign\left(\left\|s^{\prime }\right\|-\sigma^{\prime }\right)\\ \lambda^{\prime }=2w\kappa^{\prime } \end{array}\right.$$

where r>0, w>0; Meanwhile, the adjustment parameter $\sigma^{\prime }$ is selected as $\sigma^{\prime }=\alpha \left({\left\|s^{\prime }\left(t_{k}\right)\right\|}^{\frac{1}{2}}+\beta \right)/K$. Then, the triggering condition is proposed as

(60) $$t_{k+1}=\inf\left\{t:t>t_{k},\;K\left\|N^{\prime }\right\|\left\|e^{\prime }\left(t\right)\right\|\geq \alpha \left({\left\|s^{\prime }\left(t_{k}\right)\right\|}^{\frac{1}{2}}+\beta \right)\right\}$$

with $e^{\prime }\left(t\right)=E\left(t_{k}\right)-E\left(t\right)$.

Based on Theorems 1 and 2, we can present the following Lemma.

**Lemma 1.**  *Considering the system (56) under assumptions 3 and 4, and if the adaptive controller is presented as (58)–(60), the $\xi_{1}$ will be converged to the following band:*

(61) $$\left\{\xi_{1}\in R^{2}:\left\|\xi_{1}\right\|\leq \max{\left(\frac{\Omega^{2}}{\left(\rho_{\min}\left(P\right)\partial \right)}\right)}^{2},\alpha \left({\left\|s^{\prime }\left(t_{k}\right)\right\|}^{\frac{1}{2}}+\beta \right)/K\right\}$$

*where Ω and P satisfy (34) and (36), respectively, and Zeno behavior can be avoided when the triggering sequence occurs.*

**Figure 1:**
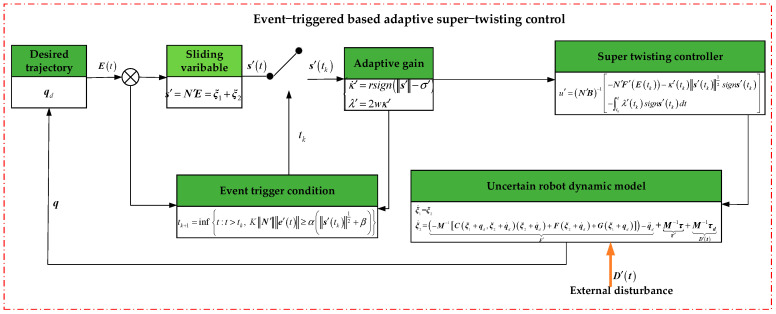
A block diagram of the proposed control scheme.

A control block diagram is shown in Figure 1.

## 5. Simulation and Experiments

**Figure 2:**
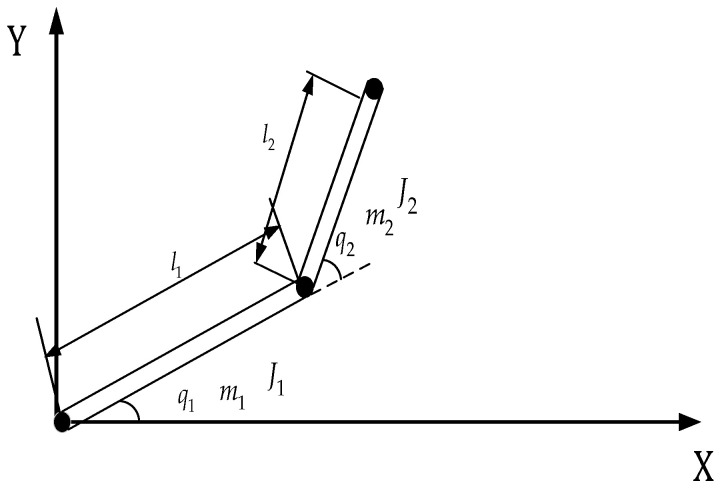
Two-degrees-of-freedom (2-DOF) robot manipulator.

To prove the effectiveness of the presented control method, simulations were carried out on the robot manipulator shown in Figure 2.

### 5.1. Model Parameters

The dynamic equation of the 2-DOF robot manipulator is given by [2]

(62) $$\begin{array}{ll} M\left(q\right)= & \left[\begin{array}{cc} m_{11} & m_{12}\\ m_{21} & m_{22} \end{array}\right],C\left(q,\dot{q}\right)\dot{q}=\left[\begin{array}{c} C_{1}\\ C_{2} \end{array}\right],G\left(q\right)=\left[\begin{array}{c} G_{1}\\ G_{2} \end{array}\right],F\left(\dot{q}\right)=\left[\begin{array}{c} f_{c1}\\ f_{c2} \end{array}\right],\tau_{d}=\left[\begin{array}{c} \tau_{d1}\\ \tau_{d2} \end{array}\right]\\ & \;\;\;\;\left\{\begin{array}{l} m_{11}=l_{2}^{2}m_{2}+2l_{1}l_{2}m_{2}\cos\left(q_{2}\right)+l_{1}^{2}\left(m_{1}+m_{2}\right)+J_{1}\\ m_{12}=m_{21}=m_{2}l_{2}^{2}+m_{2}l_{1}l_{2}\cos\left(q_{2}\right)\\ m_{22}=l_{2}^{2}m_{2}+J_{2}\\ C_{1}=-l_{1}l_{2}m_{2}\sin\left(q_{2}\right){\dot{q}}_{1}^{2}-2l_{1}l_{2}m_{2}\sin\left(q_{2}\right){\dot{q}}_{1}{\dot{q}}_{2}\\ C_{2}=l_{1}l_{2}m_{2}\sin\left(q_{2}\right){\dot{q}}_{2}^{2}\\ G_{1}=l_{2}m_{2}g\cos\left(q_{1}+q_{2}\right)+\left(m_{1}+m_{2}\right)l_{1}g\cos\left(q_{1}\right)\\ G_{2}=l_{2}m_{2}g\cos\left(q_{1}+q_{2}\right)\\ f_{c1}=0.385sign\left(\dot{q}\right)+0.726\dot{q}\\ f_{c2}=0.021\dot{q}\\ \tau_{d1}=2\sin\left(t\right)+0.05\sin\left(200\pi t\right)\\ \tau_{d2}=\cos\left(2t\right)+0.05\sin\left(200\pi t\right) \end{array}\right. \end{array}$$

The parameters of Equation (62) are listed in Table 1.

Next, the parameters of the controller were set to r=0.5, w=10, β=0.005, a=0.8, and K=3, respectively. Moreover, the desired trajectory was selected as $q_{d}\left(t\right)={\left[0.1\sin\left(t/2\right)\;0.2\sin\left(t\right)\right]}^{T}$ and the initial value was chosen to be $q\left(0\right)={\left[2\;2\right]}^{T},\;\dot{q}\left(0\right)={\left[0\;0\right]}^{T}$.

### 5.2. Simulation and Discussion

**Figure 3:**
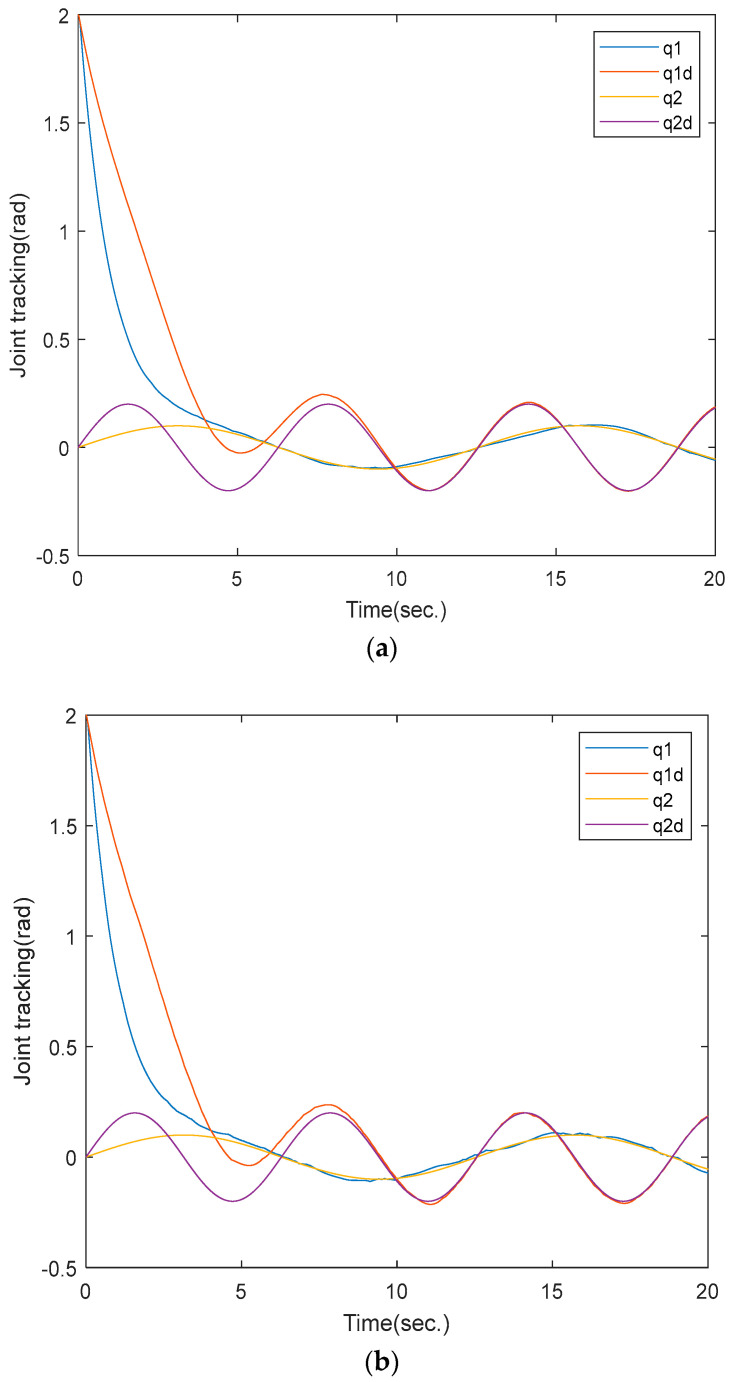
Actual ($q_{1}$, $q_{2}$) and desired ($q_{1d}$, $q_{2d}$) joint angles of the robot manipulator. (**a**) proposed method; (**b**) ETSMC scheme.

**Figure 4:**
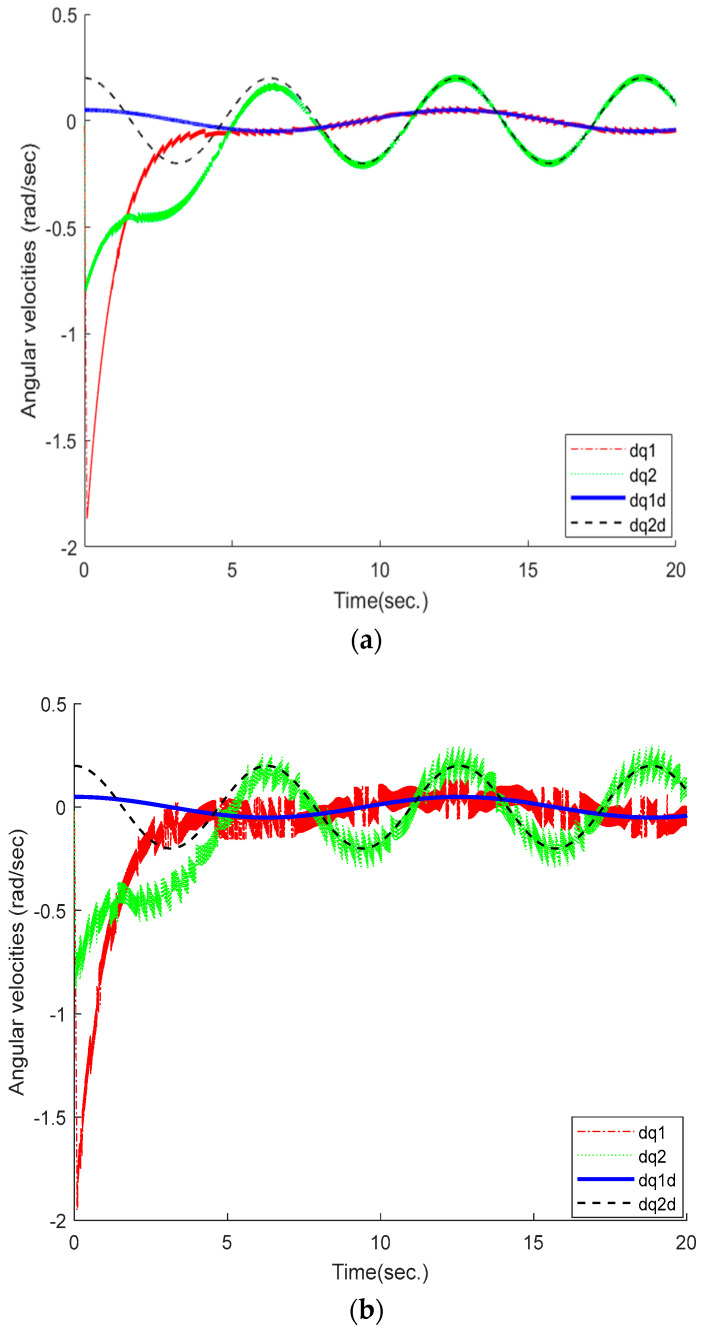
Actual ($dq_{1}$, $dq_{2}$) and desired ($dq_{1d}$, $dq_{2d}$) velocities of the robot manipulator. (**a**) proposed method; (**b**) ETSMC scheme.

**Figure 5:**
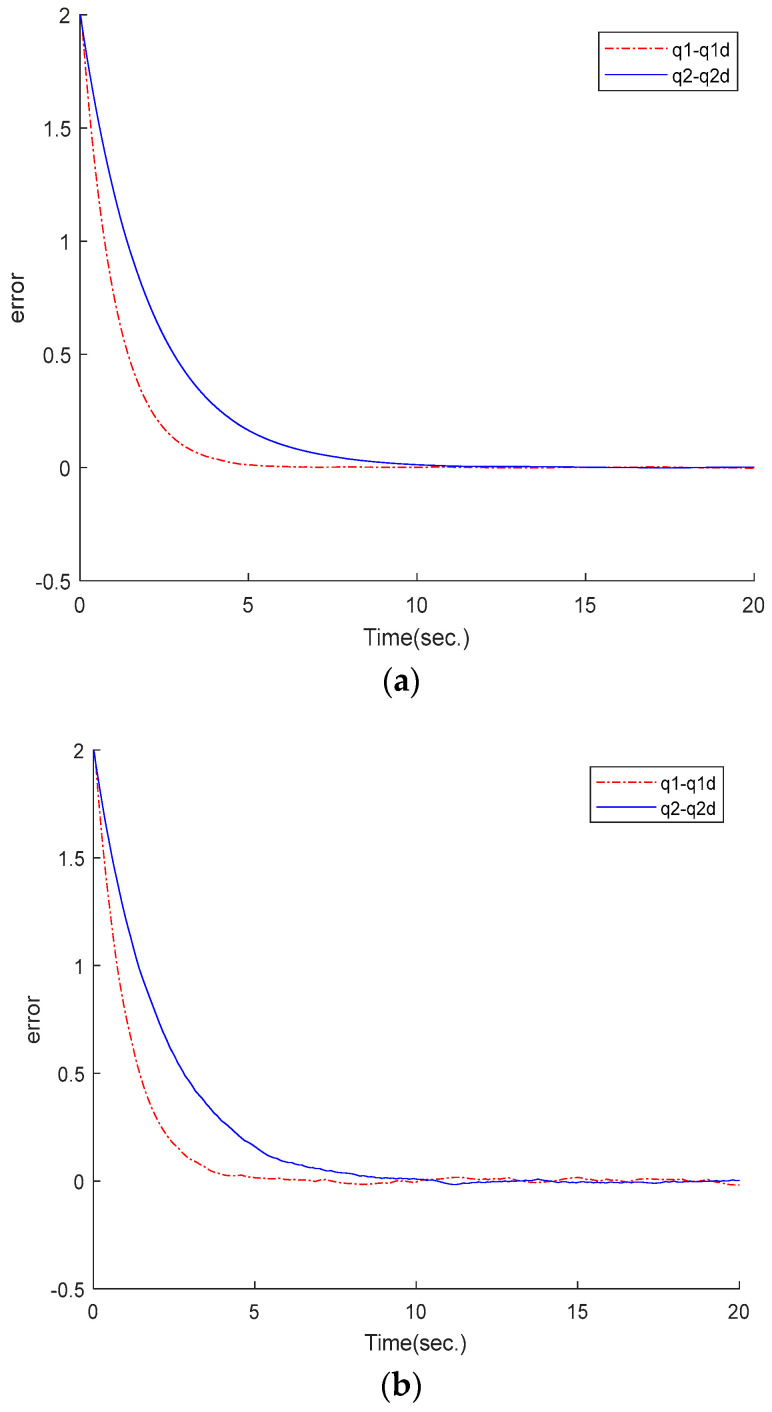
Numerical simulation: tracking error: (**a**) proposed method; (**b**) ETSMC scheme.

**Figure 6:**
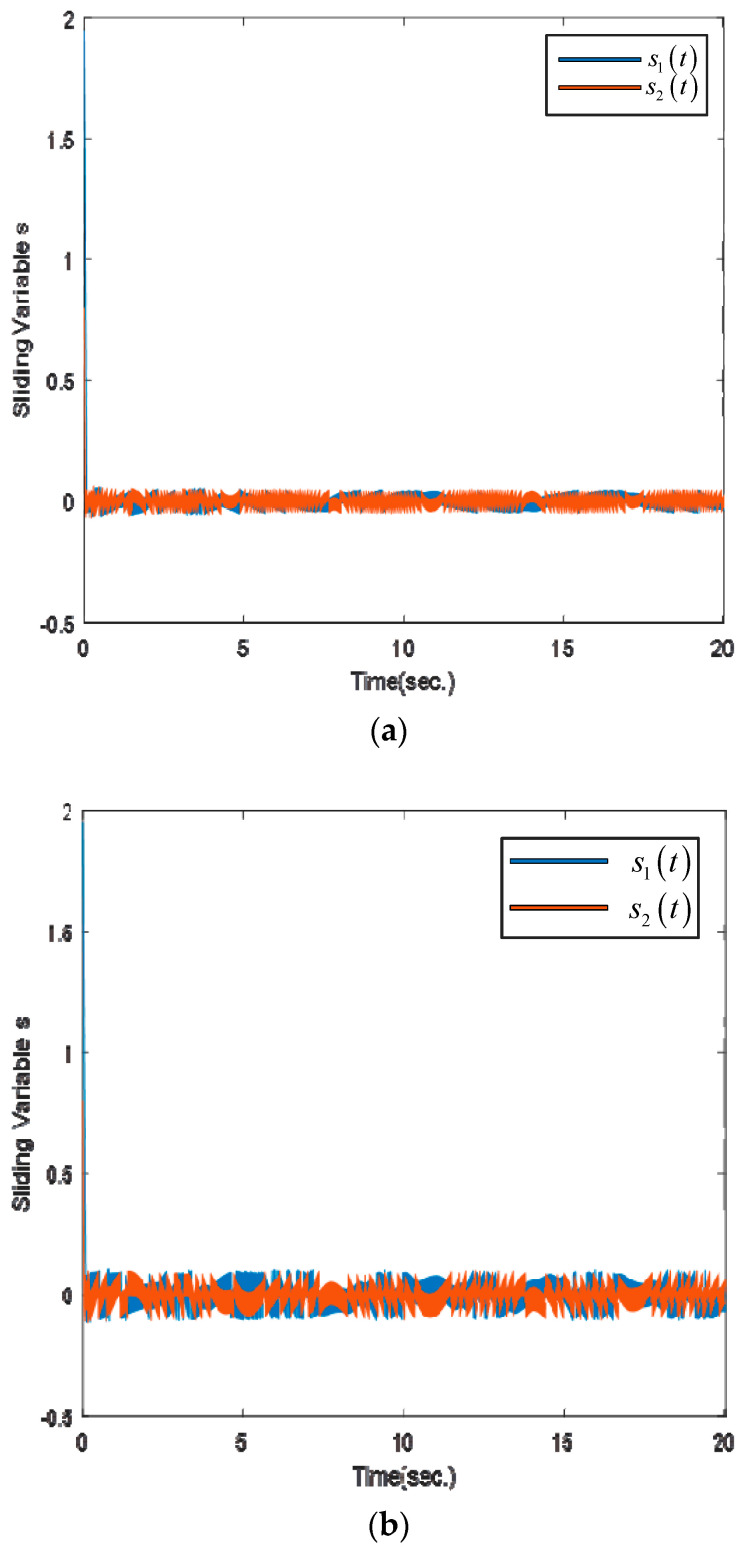
The sliding variables via numerical simulation: (**a**) proposed method; (**b**) ETSMC scheme.

**Figure 7:**
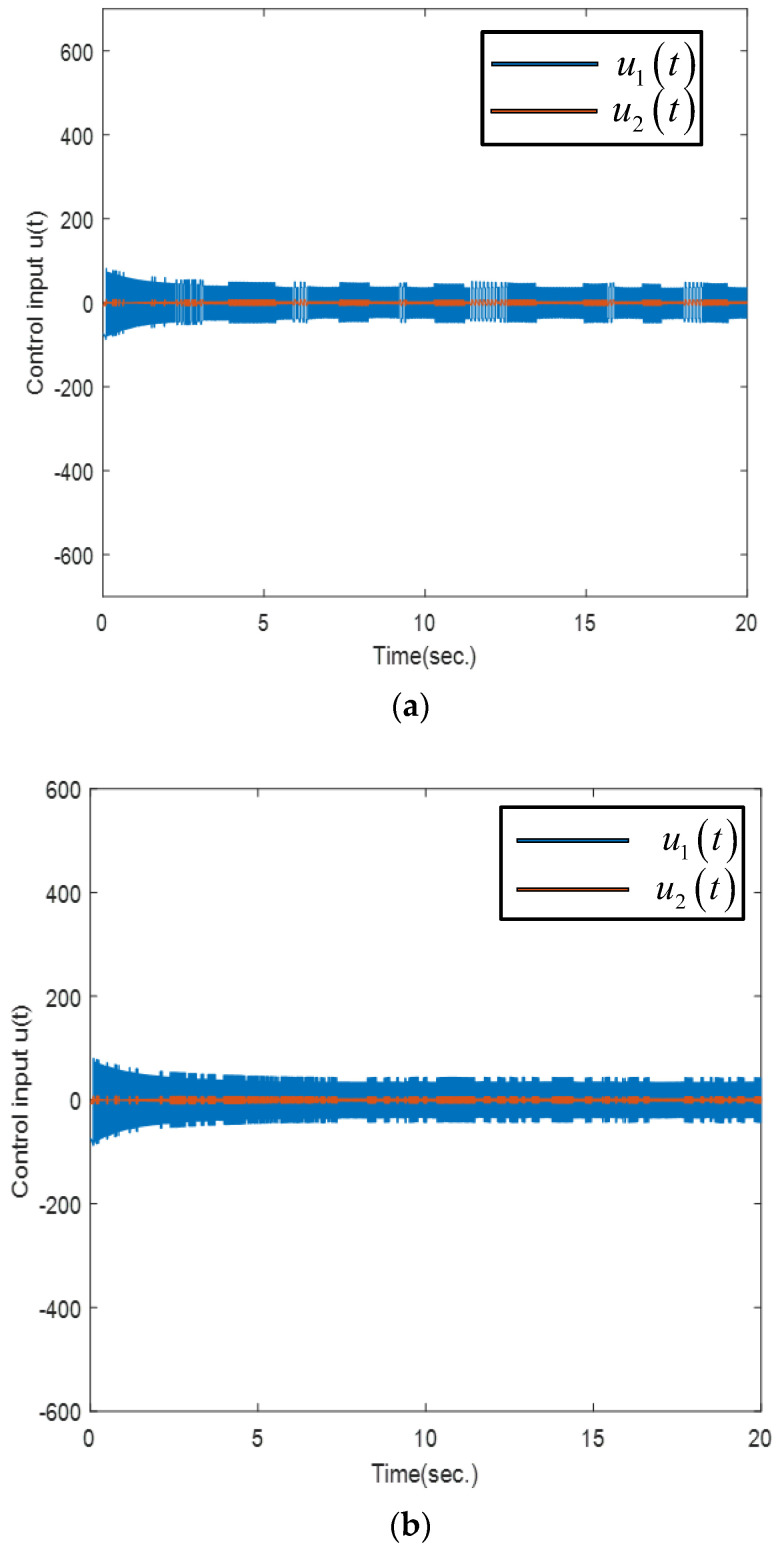
The control inputs via numerical simulation: (**a**) proposed method; (**b**) ETSMC scheme.

**Figure 8:**
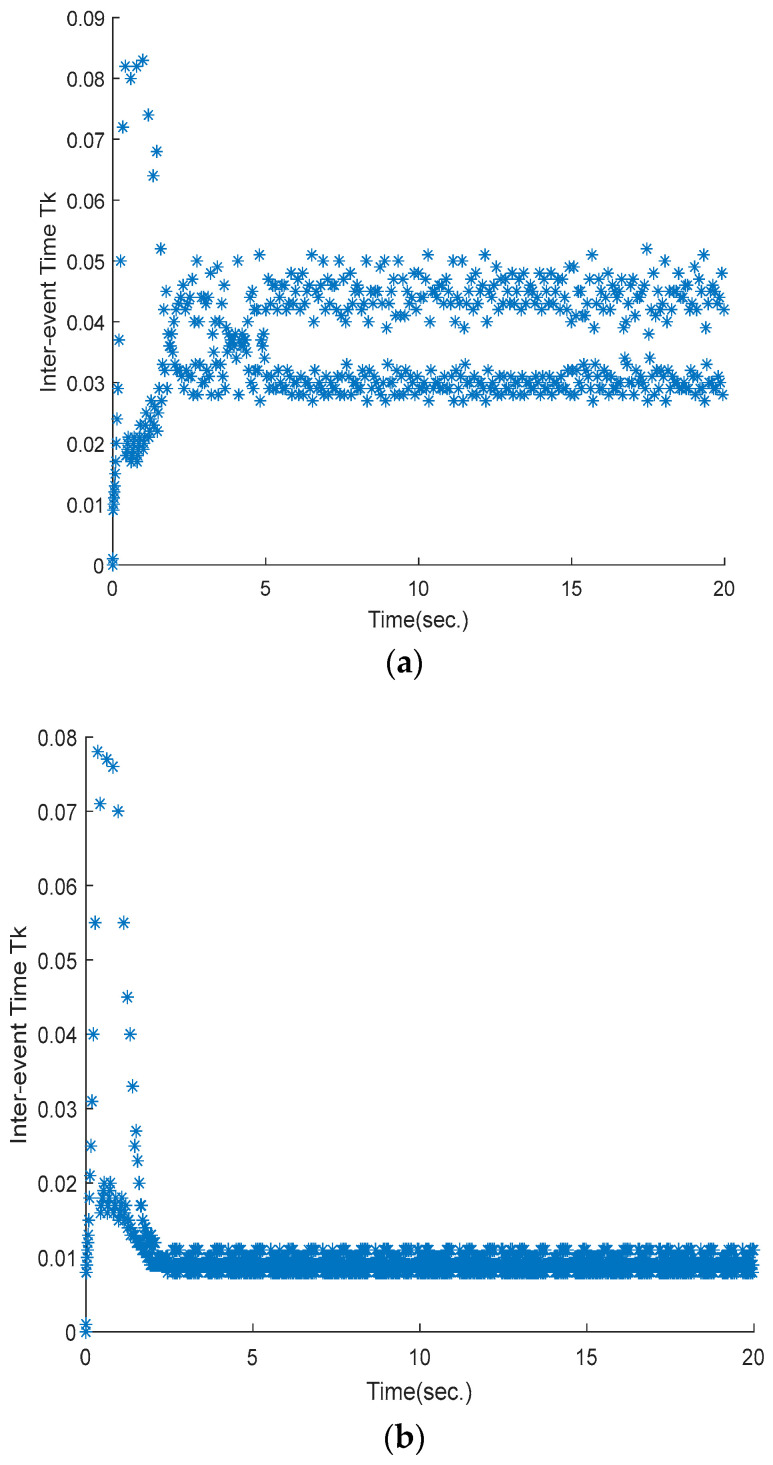
Evolution of inter-event time: (**a**) proposed method; (**b**) ETSMC scheme.

The simulation was conducted in the MATLAB (R2018b) environment. To prove the effectiveness of the designed controller, we added a comparison with the event-triggered sliding mode controller (ETSMC) [24] and conducted a detailed analysis. The numerical simulation results are demonstrated in Figure 3, Figure 4, Figure 5, Figure 6, Figure 7 and Figure 8.

The actual and desired joint angles of the robot manipulator are shown in Figure 3. As can be seen from Figure 3a, the proposed method can quickly track the desired joint angles. The simulation results of the ETSMC scheme are shown in Figure 3b. It can be seen that the ETSMC can achieve a similar tracking accuracy.

In addition, Figure 4a compares the actual and the desired joint velocities of the robot system. The results show that under the proposed controller, the desired joint velocities are quickly tracked by the actual joint velocities. Although Figure 4b also tracks the desired trajectory, the tracking curve has a relatively large jitter, which may cause system instability in severe cases.

From Figure 5a, it can be observed that the tracking error of the robot manipulator rapidly converges to the origin at 8 s. However, the ETSMC scheme fluctuates within a small range around zero in Figure 5b.

In Figure 6a, the evolution of the sliding trajectories demonstrates that the sliding variable can reach the sliding manifold in a finite time. However, the finite time of Figure 6b demonstrates that the sliding variable that reaches the sliding manifold is longer than the proposed control scheme.

The control inputs are described in Figure 7. From Figure 7a, it is apparent that the control is updated after a long time interval, which significantly reduces the computational work. In addition, from Figure 7b, the inter-event time obtained by the proposed controller is much longer than that obtained by the ETSMC scheme. Therefore, compared to the ETSMC scheme, the proposed method can effectively reduce the number of triggering instants without reducing the control accuracy, making more reasonable use of the system’s limited resources.

The inter-execution time is shown in Figure 8. It can be clearly seen from Figure 8a that the event interval time is always positive under the proposed controller. That is, it has a positive lower bound value and is not fixed. Hence, Zeno behavior can be avoided when triggering instants occur. Figure 8a also shows that the maximum value is 0.083 s, while the maximum value is 0.077 s in Figure 8b. This means that the number of triggering instants is much smaller than that obtained by the ETSMC algorithm, which decreases the error accuracy.

To verify the effectiveness of the ETASTC algorithm, we also compiled Table 2 to compare the performance of the system when it is implemented using event-triggering and periodic control. It can be seen from Table 2 that to achieve approximately the same error accuracy, such as when the error is $2.52\;\times \;10^{-2}$, the periodic implementation requires 1075 control update instants, while the event triggering implementation only needs 530. This means that compared to when implementing event-triggering, periodic control requires more control update instants, which indicates that the proposed method can save computational costs without degrading the system control performance. It should be pointed out that, as can be seen in Table 2, in the case of periodic implementation, a smaller number of control updates can be achieved by choosing a larger sampling interval. However, the accuracy of the error precision is decreased.

### 5.3. Experimental Results

**Figure 9:**
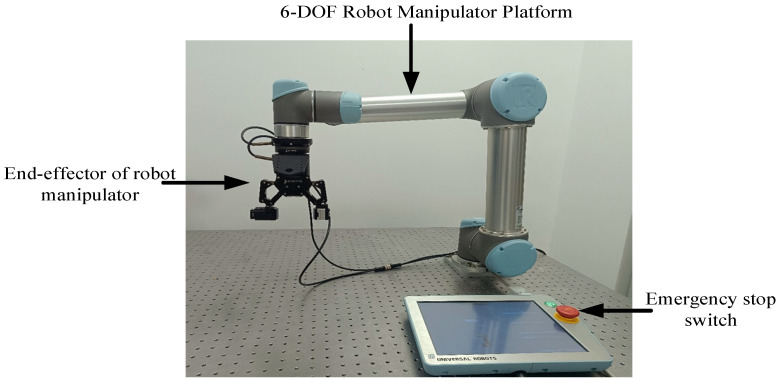
Experimental platform.

To further prove the effectiveness of the proposed controller, experiments were carried out on the UR5 robot manipulator. The layout of the experimental system is demonstrated in Figure 9. The platform mainly consists of a UR5 robot manipulator and a network communication module, aiming to achieve precise robot manipulator tracking control. UR5 is a six-degrees-of-freedom collaborative robot manipulator produced by Universal Robots, which is widely used in automated experiments, industrial operations and other fields. The robot manipulator has high flexibility and precise motion-control capabilities, making it suitable for visual servoing and other advanced control tasks. Each joint of the UR5 robot manipulator is equipped with a high-precision motor and encoder, ensuring precise positioning and motion control. The UR5 robot manipulator uses the Universal Robots controller and supports multiple interfaces, such as Ethernet, RS-232 and RS-485, and can be seamlessly connected to external computers or other devices. The maximum working radius is 850 mm, which is suitable for smaller tasks. The UR5 has a repeatability of ±0.1 mm and a maximum load of 5 kg, which can meet the needs of high-precision control.

**Figure 10:**
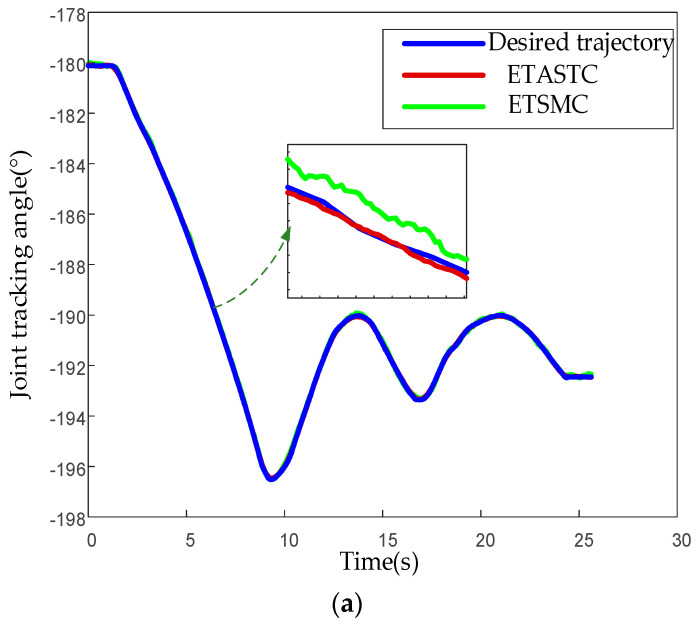

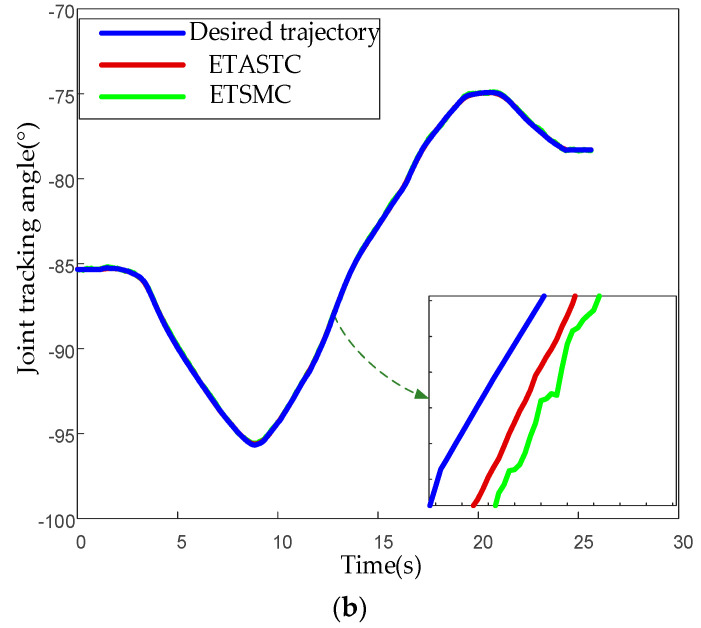

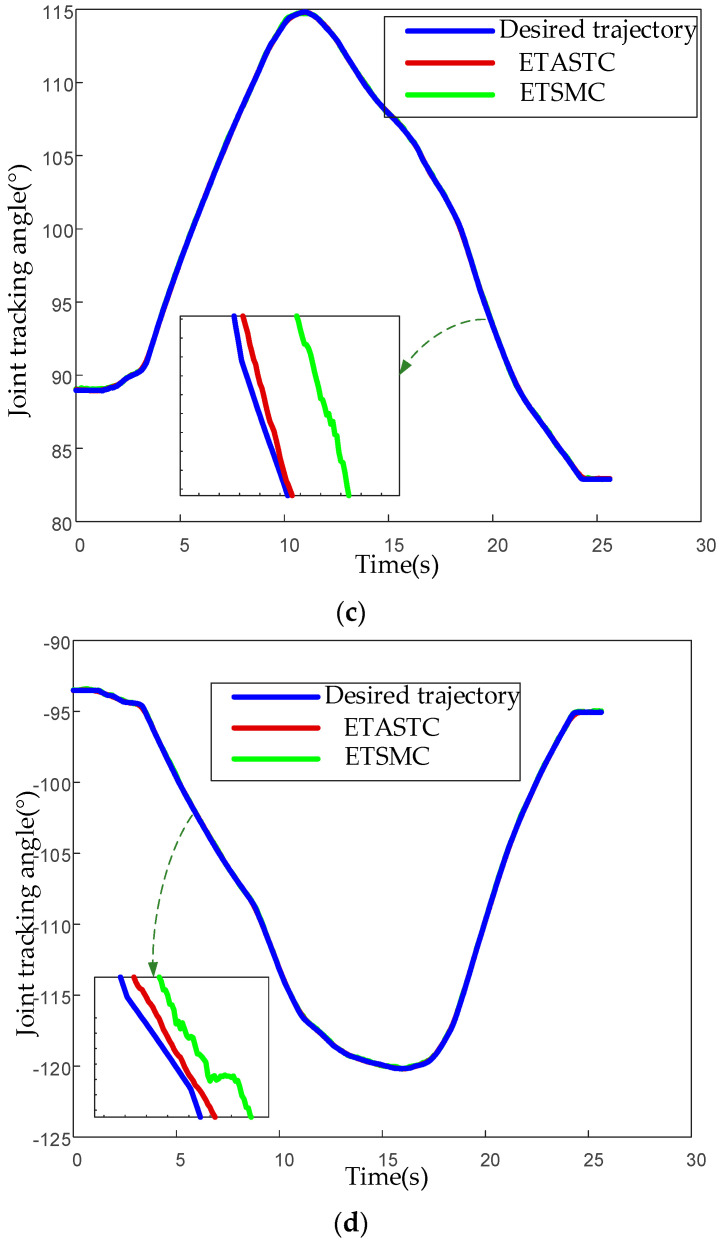

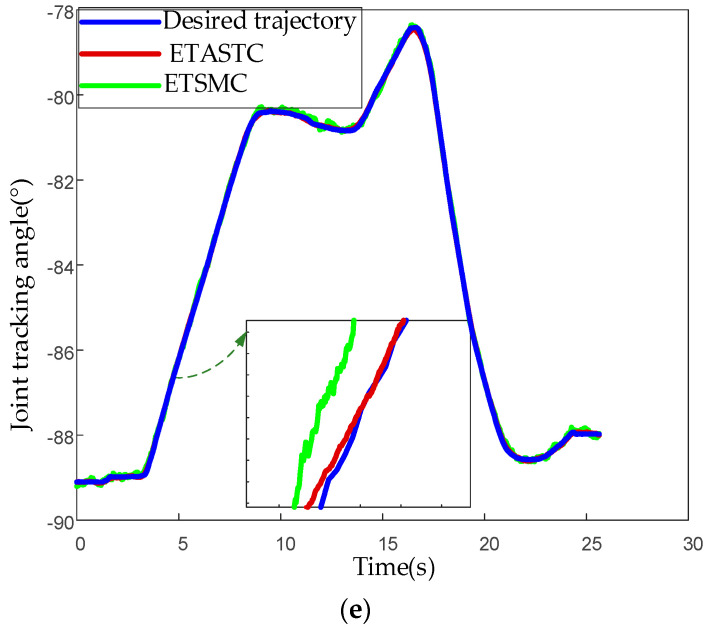

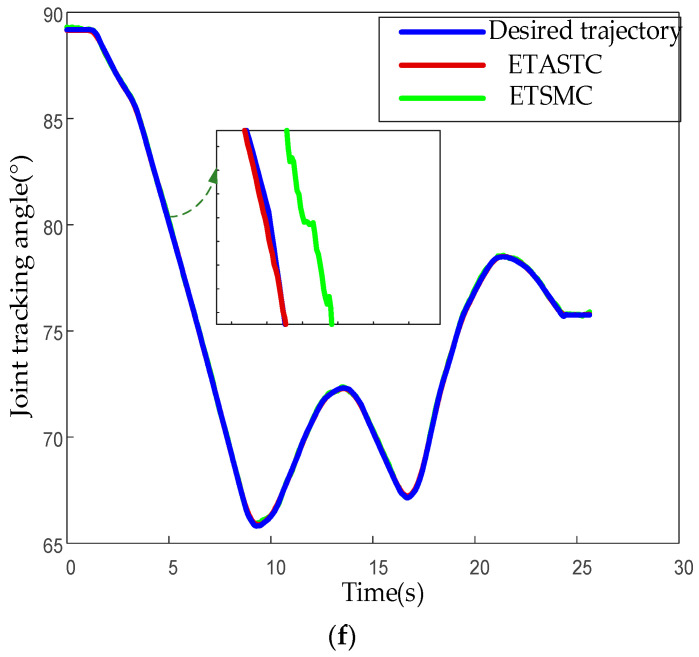
Trajectory tracking effect of the 6-DOF robot manipulator: (**a**) tracking trajectory of joint 1; (**b**) tracking trajectory of joint 2; (**c**) tracking trajectory of joint 3; (**d**) tracking trajectory of joint 4; (**e**) tracking trajectory of joint 5; (**f**) tracking trajectory of joint 6.

The initial joints of the robot manipulator values are $q_{i}=[\begin{array}{cccccc} -180.11 & -85.33 & 88.96 & -93.52 & -89.10 & 89.18 \end{array}]$ and ${\dot{q}}_{i}=\left[\begin{array}{cccccc} 0 & 0 & 0 & 0 & 0 & 0 \end{array}\right]$. The settings of the other parameter values were the same as in the previous section. In this experiment, we employed an event-triggered control mechanism, where control signals are updated only when the system state exceeds the preset trigger threshold, thereby reducing unnecessary computational calculations. The UR5 robot performed a series of dynamic trajectories (such as multiple reciprocating motions). During the motion process, the system status was continuously fed back to the controller, and the control signal was updated only when the error or status satisfied the trigger condition. This mechanism effectively saved computing resources while ensuring the stability and accurate tracking of the system. The experimental results of the two control algorithms are shown in Figure 10.

From the enlarged part in Figure 10a–f, it can be seen that the tracking trajectory of the ETSMC algorithm has obvious lag (especially in the initial 5 s) and steady-state oscillation, and the maximum angle deviation is greater than 3°. However, the actual trajectory of the proposed ETASTC algorithm closely follows the desired trajectory, with a fast dynamic response, a steady-state error less than 0.5°, and no obvious chattering. The ETSMC control scheme responds slowly, while the ETASTC method converges quickly to the desired trajectory. ETSMC exhibits periodic fluctuations, while ETASTC maintains smooth tracking, which verifies the superiority of the proposed algorithm in tracking the trajectory of multi-joint robot manipulators. To further demonstrate the advantages of the ETASTC algorithm, we provide the mean absolute error (MAE) results of the tracking error for each joint. The MAE formula can be expressed as

(63) $$MAE=\frac{1}{N}\sum_{k=1}^{N}\left|q_{desired}\left(k\right)-q_{actual}\left(k\right)\right|$$

where $q_{desired}\left(k\right)$ and $q_{actual}\left(k\right)$ represent the desired joint angle and the actual joint angle, respectively. A comparison of the MAE of the two algorithms (ETASTC and ETSMC) in each joint is listed in Table 3.

**Figure 11:**
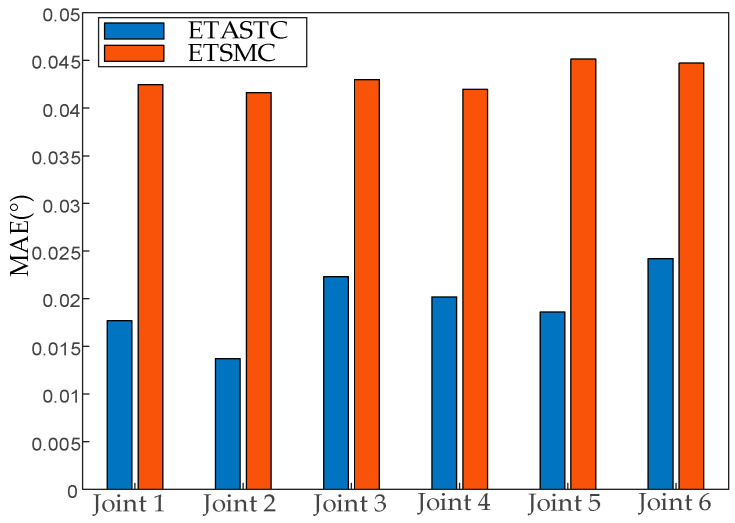
Trajectory tracking MAE results under the two control algorithms.

It can be seen from Table 3 that the MAE values of all joints of the ETASTC algorithm are significantly lower than those of ETSMC. The errors of the ETASTC algorithm for joints 1 and 6 are reduced by 58.5% and 45.9% compared to that of ETSMC, respectively. The average MAE error of the six joints shows that the proposed ETASTC algorithm is 54.9% more accurate than the comparison algorithm ETSMC. The MAE range of ETASTC (0.0137°–0.0242°) is narrower than that of ETSMC (0.0416°–0.0451°), indicating that its control strategy is more robust on different joints. By dynamically adjusting the sliding mode gain and disturbance compensation term, ETASTC effectively suppresses the chattering problem of the traditional sliding mode, thus reducing the MAE. The MAE results of the trajectory tracking of the different algorithms on a 6-DOF robot manipulator are shown in Figure 11.

As can be seen from Figure 11, the error column heights of each joint of the proposed ETASTC algorithm are uniform and low, indicating that the algorithm is more adaptable to multi-DOF coupled dynamics. However, the error column heights of the comparison algorithm ETSMC are generally high and fluctuate less, which may be due to the fixed gain of the terminal sliding mode control, which leads to limited global performance. The proposed algorithm can realize a decentralized control strategy for the coupling effect of robot joints to ensure the balance of errors in each joint.

## 6. Conclusions

In this paper, an event-triggered control strategy combined with adaptive super-twisting for uncertain robot manipulator system was presented. The designed controller is robust to unknown external disturbances and uncertainties and does not require the upper bound values of these disturbances and uncertainties. Moreover, the proposed control scheme guarantees the convergence of inter-sampling behavior and ensures the stability of uncertain robot manipulator systems. Additionally, the system exhibits Zeno-free execution, as demonstrated through rigorous analysis. The numerical results showed that the presented control method achieves a satisfactory control performance while saving the system computing resources. Therefore, the performance of uncertain robot manipulator systems can be significantly improved in terms of the number of control updates. In our future work, research on robot manipulators in the medical field is a potential topic, including the design and kinematics analysis of transluminal endoscopic surgery [29] and the design of positioning systems for endoscopic microsurgery [30].

## Figures and Tables

**Table 1:** The main parameters of the 2-DOF robot manipulator.

Symbol	Definition	Value
$m_{1}$	Nominal mass of link 1	0.4 kg
$m_{2}$	Nominal mass of link 2	1.2 kg
$l_{1}$	Length of link 1	1.0 m
$l_{2}$	Length of link 2	0.85 m
$J_{1}$	Inertia tensor of link 1	2 kg·m^2^
$J_{2}$	Inertia tensor of link 2	2 kg·m^2^
g	Gravity acceleration	9.8 m/s^2^

**Table 2:** System performance for the different control schemes.

Implementation Technique	Number of Control Updating Instants	$\mathrm{Error}\;\mathrm{Accuracy}\;(q_{1},q_{2}$)
Event triggering (β = 0.005)	530	$2.52\;\times \;10^{-2}$
Periodic control (τ = 0.002s)	1075	$2.53\;\times \;10^{-2}$
Periodic control (τ = 0.004s)	830	$2.66\;\times \;10^{-2}$

**Table 3:** Comparison of the MAE results of the two algorithms.

Degrees of Freedom	ETASTC	ETSMC
Joint 1	0.0176	0.0424
Joint 2	0.0137	0.0416
Joint 3	0.0223	0.0429
Joint 4	0.0201	0.0419
Joint 5	0.0186	0.0451
Joint 6	0.0242	0.0447
Mean	0.0194	0.0430

## Data Availability

The data that support the findings of this study are included within the article.
